# Proximal Hyperspectral Imaging Detects Diurnal and Drought-Induced Changes in Maize Physiology

**DOI:** 10.3389/fpls.2021.640914

**Published:** 2021-02-22

**Authors:** Stien Mertens, Lennart Verbraeken, Heike Sprenger, Kirin Demuynck, Katrien Maleux, Bernard Cannoot, Jolien De Block, Steven Maere, Hilde Nelissen, Gustavo Bonaventure, Steven J. Crafts-Brandner, Jonathan T. Vogel, Wesley Bruce, Dirk Inzé, Nathalie Wuyts

**Affiliations:** ^1^Department of Plant Biotechnology and Bioinformatics, Ghent University, Ghent, Belgium; ^2^VIB-UGent Center for Plant Systems Biology, Ghent, Belgium; ^3^BASF SE, Ghent, Belgium; ^4^BASF Corporation, Research Triangle Park, NC, United States

**Keywords:** automated phenotyping platform, hyperspectral, phenotyping, drought, physiology, maize, proximal sensing

## Abstract

Hyperspectral imaging is a promising tool for non-destructive phenotyping of plant physiological traits, which has been transferred from remote to proximal sensing applications, and from manual laboratory setups to automated plant phenotyping platforms. Due to the higher resolution in proximal sensing, illumination variation and plant geometry result in increased non-biological variation in plant spectra that may mask subtle biological differences. Here, a better understanding of spectral measurements for proximal sensing and their application to study drought, developmental and diurnal responses was acquired in a drought case study of maize grown in a greenhouse phenotyping platform with a hyperspectral imaging setup. The use of brightness classification to reduce the illumination-induced non-biological variation is demonstrated, and allowed the detection of diurnal, developmental and early drought-induced changes in maize reflectance and physiology. Diurnal changes in transpiration rate and vapor pressure deficit were significantly correlated with red and red-edge reflectance. Drought-induced changes in effective quantum yield and water potential were accurately predicted using partial least squares regression and the newly developed Water Potential Index 2, respectively. The prediction accuracy of hyperspectral indices and partial least squares regression were similar, as long as a strong relationship between the physiological trait and reflectance was present. This demonstrates that current hyperspectral processing approaches can be used in automated plant phenotyping platforms to monitor physiological traits with a high temporal resolution.

## Introduction

One of the major challenges in plant biology and crop research is elucidating the link between genome, physiological processes, plant trait performance, and yield across genotypes and environments. This requires combining vast amounts of genotypic data with corresponding phenotypic measurements (Yang et al., 2014; Clauw et al., 2016). To facilitate the collection of phenotypic measurements, high-throughput phenotyping platforms have been developed to automate data collection on a large number of plants (Granier et al., 2006; Busemeyer et al., 2013; Virlet et al., 2017). These platforms are often equipped with imaging systems that capture data non-destructively and monitor plant features over time (Humplík et al., 2015).

One imaging technology that has gained popularity in recent years is hyperspectral imaging, which can record high spectral resolution data of the visible and near-infrared (VNIR) and shortwave-infrared (SWIR) regions of the electromagnetic spectrum. Depending on the spectral range of these sensors, a wide variety of physiological traits can be studied, such as photosynthetic efficiency and pigment content (Inoue et al., 2008; Yendrek et al., 2017), leaf thickness (Neilson et al., 2015), water (Kim et al., 2015), nitrogen (Serrano et al., 2002; Yendrek et al., 2017), and lignin (Serrano et al., 2002) content. This wide range of physiological relationships has resulted in the use of hyperspectral sensors for research areas including disease detection (Huang et al., 2007), drought stress (Römer et al., 2012), pigment content (Yendrek et al., 2017), photosynthetic activity (Yendrek et al., 2017; Fu et al., 2019), and yield (Zhang and He, 2013). Hyperspectral studies have been performed on both landscape level (remote sensing), and plant or organ level (proximal sensing). Proximal sensing has been applied on both field and in-door phenotyping studies by mainly using non-imaging spectrographs (Odilbekov et al., 2018; Ge et al., 2019; Osco et al., 2020). More recent studies on the other hand have utilized hyperspectral imaging for close-range phenotyping on in-door automated platforms, allowing the monitoring of spatial and temporal variations in traits that were previously inaccessible (Ge et al., 2016; Moghimi et al., 2018; Thomas et al., 2018).

In-door automated phenotyping platforms provide a more controlled phenotyping environment and higher spatial resolution compared to remote sensing and close-range field setups. This difference in experimental setup may influence the effectiveness of common hyperspectral processing approaches, such as indices and machine learning algorithms. Indices are commonly used in remote sensing to reduce the voluminous multidimensional data, which can be challenging to analyze. Some hyperspectral indices have been used in in-door phenotyping studies to detect the effects of extreme temperature and salinity stress (Simko et al., 2016; Feng et al., 2018). Many hyperspectral indices have been developed though on data collected during severe biotic or abiotic stresses to increase the measurement range that can be linked to reflectance (Gamon et al., 1992; Peñuelas et al., 1993; Gitelson and Merzlyak, 1997; Gitelson et al., 2002). Consequently, these indices perform well in severe stress studies, while it is uncertain how sensitive they are to more subtle physiological differences, which can be more easily investigated under controlled conditions. More recent studies have demonstrated the use of machine learning algorithms to analyze hyperspectral data collected in in-door phenotyping platforms. These algorithms can learn the relationship between the plant spectrum and a trait in an automated manner. Several algorithms, such as support vector machines, simplex volume maximization (Thomas et al., 2018), artificial neural networks (Kong et al., 2014) and partial least square regression (PLSR; Pandey et al., 2017), have been used successfully to predict phenotypic traits or classify different degrees of stress.

The aforementioned methods are also affected by the higher resolution in proximal in-door phenotyping setups, as the effect of plant geometry on illumination and reflectance variation is more pronounced. This additional non-biological reflectance variation can mask biological effects and complicate hyperspectral data interpretation. Several methods, such as 3-dimensional (3D) modeling and standard normal variate normalization, have been proposed to reduce this variation (Vigneau et al., 2011; Behmann et al., 2016; Roscher et al., 2016; Asaari et al., 2018). These approaches require, however, additional 3D information or perform transformations, which may complicate data interpretation by limiting the use of indices (Asaari et al., 2018). An alternative and more intuitive method to reduce illumination effects is to subdivide plant pixels into sun-lit and shaded classes. The effectiveness of this approach has been demonstrated in remote sensing (Clark et al., 2005), whereas its usefulness in in-door automated phenotyping platforms is not yet established. In this study, the performance of this alternative approach to reduce the more pronounced illumination effects in proximal sensing was examined by performing a light classification on the plant pixels.

Besides examining the non-biological reflectance variation associated with a proximal imaging setup, the use of spectral measurements for proximal sensing and its application with regard to diurnal, developmental and drought-induced responses were investigated in an automated high-throughput phenotyping system. The prediction accuracies of vegetation indices and PLSR models were compared in order to determine whether common hyperspectral data analysis approaches could provide accurate proxies for physiological plant traits. The case study chosen for this analysis was the effect of drought stress, i.e., soil water deficit and high vapor pressure demand, on the reflectance and physiology of maize. The experiments focused on moderate drought stress that allowed maize plants to continue their growth and development at a reduced rate without experiencing senescence.

## Materials and Methods

### Experimental and Hyperspectral Imaging Setup in the PHENOVISION Automated Plant Phenotyping System

PHENOVISION is a phenotyping platform located in the greenhouse infrastructure of the VIB-UGent Center for Plant Systems Biology (Ghent, Belgium), which is primarily used for maize phenotyping. The platform allows the monitoring of 392 plants throughout their vegetative development by non-destructive imaging.^1^ It consists of a conveyor belt system that transports plants from the growth area to the automated weighing-irrigation stations and imaging cabins. These imaging cabins contain three camera systems: a red-green-blue camera system in a multi-view setup for growth-related phenotyping, a thermal infrared camera for estimating plant water use behavior, and a proximal top-view hyperspectral imaging system for physiological phenotyping. The hyperspectral imaging system consists of two pushbroom line scanner spectrographs (VNIR and SWIR) mounted on a motorized linear stage (1.5 m in length) in a dedicated cabin that is equipped with a white reference surface, halogen lighting frames that move alongside the cameras, and a lift with rotating platform, which positions the plant at the optimal distance from the top-view cameras and at the level of the white reference surface. The spectrographs possess a spectral range of 400–1,000 nm (ImSpector V10E, SPECIM, Finland) and 970–2,500 nm (ImSpector N25E, SPECIM, Finland). The spectral resolution of the VNIR V10E is 0.72 nm. A spectral binning of four was applied, reducing the resolution to 3 nm (194 bands). The V10E contains 1312 pixels per line, which were binned by four to match the SWIR spectrograph producing 328 pixels. The SWIR N25E has a spectral resolution of 6.3 nm (256 bands) and contains 320 pixels per line. In total, 511 lines were scanned to create an image of 511 × 328 or 320 pixels. The focal lengths of the V10E and N25E are 18.5 and 15 mm, respectively. Both spectrographs have a field of view of 0.75 m and a spatial resolution of 2.35 mm at 1.2 m, which is the optimal distance from the scanners regarding focal depth. The spectral data acquired by the line scanners consists of the aerial portion of a single plant, the white reference surface, and a black reference (camera shutter closed).

Hyperspectral imaging in PHENOVISION has been optimized for high-throughput phenotyping with data acquisition occurring at a rate of one minute per plant (time between the entrance and exit of each plant in the imaging cabin).

For this study, two drought stress experiments were performed: an exploratory experiment (EXP) that investigated the effect of drought on reflectance, and a validation experiment (VAL) in which the drought-induced changes in reflectance were linked to physiological traits. Maize plants were grown in a semi-controlled environment in which air temperature was set to 22–23°C and vapor pressure deficit (VPD) to 1–1.2 kPa by means of air relative humidity modifications until the V5 stage of maize vegetative development, i.e., during seeding establishment. After the V5 stage, the environment system was adjusted to provide a diurnal gradient, with the temperature gradually changing from 22°C at night to 28°C in the afternoon. This diurnal temperature pattern resulted in diurnal variations in VPD of 0.95–2 kPa (night/afternoon), attempting to mimic diurnal variations in temperature and VPD under field conditions. A 16/8-h day/night light cycle was implemented in the greenhouse using high-pressure sodium vapor lamps, to achieve an average light intensity of 280 μmol m^–2^ s^–1^. The greenhouse photosynthetically active radiation (PAR), relative humidity, VPD and temperature were continuously monitored by four environmental monitoring stations containing an SKH 2053 humidity and temperature sensor, and a PAR SKL 2625 sensor (Skye Instruments, United Kingdom). Maize (*Zea mays* L.) inbred B104 (Liu et al., 2003) plants were sown in 7-l pots filled with 850 g of peat-based soil with osmocote fertilizer (N.V. Van Israel, Belgium) and were randomly placed on the PHENOVISION platform. The plants were fertilized weekly with 40 ml of 200 ppm N Peters Excel CalMag Grower (Everris, Netherlands) solution once the V5 stage was reached.

During the two experiments, maize plants were monitored from emergence until the V7 stage (Ritchie et al., 1996). Plants were randomly subdivided in three groups: well-watered (WW), water deficit (WD) and border plants. EXP included 77 WW, 76 WD, and 80 border plants, while VAL had 82 WW, 77 WD, and 68 border plants. In both experiments, the plants were irrigated to a WW soil water content (WC) of 2.4 g H_2_O g^–1^ dry soil (soil water potential of –10 kPa) until plants reached the V5 stage, after which the drought treatment was initiated. Water was withheld from WD plants for 6–7 days (acute drought period) until a soil WC of 1.4 g H_2_O g^–1^ dry soil (soil water potential of –100 kPa) was attained, after which plants were irrigated to sustain the WD soil WC. The end of the acute drought period was reached at the V6–V7 stage (six to seven fully developed leaves). Throughout both experiments, hyperspectral images were collected, while physiological measurements using standard measurement equipment were only obtained during the drought period of VAL.

### Physiological Measurements

Non-destructive physiological measurements were collected on day 0, 5, 7, and 9 of the drought treatment period, while one additional day (day 3) was added for the destructive measurements. Five WW and five WD plants were selected for non-destructive measurements and monitored at four different time points (8:00, 11:00, 14:00, and 17:00) on days 0 and 5, and at five time points (8:00, 11:00, 13:00, 15:00, and 17:00) on days 7 and 9. The destructive measurements were collected three times a day (9:00, 12:30, and 16:30) on three to five WW and WD plants.

The non-destructive measurements consisted of photosynthetic rate (A), transpiration rate (E), stomatal conductance to H_2_O (g_*s*_), and quantum yield based on CO_2_ (Φ_*CO*2_), effective quantum yield (Φ_*PS*2_) and energy harvesting efficiency by oxidized PS2 (F_*v*_′/F_*m*_′) and were collected with a portable LICOR 6400-XT Infrared Gas Analyzer (LI-COR Biosciences, United States). Within the leaf chamber, a steady-state CO2 level of 400 μmol^–1^ was maintained, while temperature and PAR were adjusted to the greenhouse temperature (25–31°C) and PAR (230–360 μmol photons m^–2^ s^–1^) at every measurement time point. The fluorescence parameters were determined using the LI-6400 manual guidelines for light-adapted leaves.

Destructive sampling was performed on top leaves (leaves 5–9), which were visible in the hyperspectral image. The tip of the leaf was used for water potential (Ψ) measurements collected with a pressure chamber (PMS Instrument Company, United States), whereas 5 cm from the middle of the leaf was weighed and dried to estimate WC per g dry weight. Leaf disks were sampled to measure anthocyanin, carotenoid and chlorophyll content (Supplementary Materials and Methods 1).

### Hyperspectral Data Collection and Pre-processing

Hyperspectral imaging was performed once per day between 8 AM and 3 PM in the EXP, whereas during the VAL, plants were imaged four to five times on sampling days and three times on regular days (8 AM–7 PM). The number of plants imaged per hour was ±16 for EXP and ±50 for VAL. All of the image processing was performed using the computing environment R version 3.2.2 (R Core Team, 2015) and was limited to the first 10 days after the initiation of the drought treatment (the acute drought period). The reflectance (ρ) of each image was calculated based on a white and a dark reference image which were acquired just before the scanning of the plant. The aim was to compensate for pixel-to-pixel sensor response differences and spatial non-uniformities in illumination.


$$\text{reflectance}=\frac{\text{image}-\text{dark}}{\text{white}-\text{dark}}$$

Visible and near-infrared pixels of the plants were extracted from the images using the red-edge normalized vegetation index, which has a range from 0.2 to 0.9 for green vegetation. Pixels with an index value higher than 0.35 were classified as plant pixels. The segmentation of the plant in the SWIR images was performed using a random forest model that was trained on images from juvenile (V2) to mature (VT) maize plants. The random forest model was created with the “randomForest” package for R (Liaw and Wiener, 2002) and had a confusion matrix accuracy of 99.8% on an independent test dataset. The amount of segmented plant pixels that actually contained plant was 99.6%.

### Reducing Non-biological Variation Caused by Inhomogeneous Illumination

Plant reflectance varied strongly within the plant image. This variation was typically non-biological as it resulted from inhomogeneous illumination of the plant surface. To reduce this variation, a light classification was performed that subdivided plant parts into classes with similar illumination. For the VNIR classification, brightness was used as a proxy for illumination and was calculated from blue (492 nm), green (539 nm), and red (651 nm) images using the “rgb2hsv” function of the “grDevices” R package. The brightness calculated with this function turned out to correspond with green reflection, which was the least affected by the drought treatments. Light classes were created by performing k-means clustering using the “stat” R package on the brightness values of a training dataset that included WW and WD plants from different developmental stages (V5–VT). This resulted in three classes: low, intermediate, and high light. Because the low and high-light class still showed a high variability, they were further subdivided with k-means clustering into extremely low, low, high, and extremely high light classes. This additional k-means clustering step removed most of the leaf edge and vein pixels from the low and high-light classes. The classified training dataset was used to determine fixed brightness thresholds, by looking at the distribution and overlap in brightness values of the light classes that were later applied on the whole dataset. The thresholds were used to create binary images for all the light classes of each plant. To assure that the classification of SWIR corresponded to that of VNIR, a co-registration of the VNIR binary images to the SWIR scanner was performed. The positions of VNIR pixels in the SWIR image were calculated using sample and line transformation formulas, which were developed by determining the linear relationship between the coordinates of corresponding chessboard points. After the classification, an average reflectance value was calculated for each plant–light class combination. Only the data of the intermediate light class were further analyzed in this case study. This light class was selected based on both the percentage of pixels per plant it contained and the ability to detect drought-related effects. The intermediate light class also included the physiological measurement locations within the plant.

### Indices

The development of indices and models, as well as a part of the statistics, were performed using the computing environment R version 3.4.3 (R Core Team, 2017). Publicly available indices were calculated from plant reflectance (Table 1) and their performance to detect drought and to predict physiological traits was evaluated. Additionally, new indices were created from the validation experiment (Table 2) by means of two methods. These methods used all available noise-free wavelengths (480–2470 nm) present in the hyperspectral dataset. The first method calculated all possible ratios and normalized difference ratios of wavelengths and selected the one with the highest correlation (“stats” and “rmcorr” R package) to the trait of interest (Bakdash and Marusich, 2018). The second method computed correlations for each wavelength and determined the wavelength with the highest correlation. This wavelength was subsequently combined with other wavelengths using basic mathematical operations, until the correlation between the index and trait stabilized or until the index contained four different wavelengths.

**TABLE 1:** List of published indices that were evaluated.

Index	Formula	Wavelengths (nm)	References
Modified Chlorophyll Absorption Ratio Index (MCARI)	$\text{MCARI}=\left[\left(\rho_{700}-\rho_{670}\right)-0.2\left(\rho_{700}-\rho_{550}\right)\right]*\left(\frac{\rho_{700}}{\rho_{670}}\right)$	ρ_700_ = 699 ρ_670_ = 670 ρ_550_ = 551	Daughtry et al., 2000
Carotenoid Reflectance Index 1 (CRI1)	$\text{CRI}1=\frac{1}{\rho_{510}}-\frac{1}{\rho_{550}}$	ρ_510_ = 511 ρ_550_ = 551	Gitelson et al., 2002
Red Green Ratio Index (RGRI)	$\text{RGRI}=\frac{\rho_{\text{red}}}{\rho_{\text{green}}}$	ρ_*red*_ = 600–700 ρ_*green*_ = 500–600	Gamon and Surfus, 1999
Normalized Difference Vegetation Index (NDVI)	$\text{NDVI}=\frac{\rho_{\text{NIR}}-\rho_{\text{red}}}{\rho_{\text{NIR}}+\rho_{\text{red}}}$	ρ_*NIR*_ = 800–900 ρ_*red*_ = 600–700	Rouse et al., 1974
Moisture Stress Index (MSI)	$\text{MSI}=\frac{\rho_{1599}}{\rho_{819}}$	ρ_1599_ = 1,550–1,650 ρ_819_ = 760–900	Hunt and Rock, 1989
Water Band Index (WBI)	$\text{WBI}=\frac{\rho_{970}}{\rho_{900}}$	ρ_970_ = 969 ρ_900_ = 901	Peñuelas et al., 1993
Photochemical Reflectance Index (PRI)	$\text{PRI}=\frac{\rho_{531}-\rho_{570}}{\rho_{531}+\rho_{570}}$	ρ_531_ = 532 ρ_570_ = 570	Peñuelas et al., 1995
Ratio Vegetation Index 870/610 (RVI_870/610_)	${\text{RVI}}_{870/610}=\frac{\rho_{870}}{\rho_{610}}$	ρ_870_ = 865–875 ρ_610_ = 605–615	Zhu et al., 2008
Ratio NIR/510 (R_775/510_)	${\text{R}}_{775/510}=\frac{\rho_{775}}{\rho_{510}}$	ρ_775_ = 750–800 ρ_510_ = 511	Gitelson et al., 2002
Relative Water Content index (RWC)	$\mathrm{RWC}=\frac{\rho_{1430}}{\rho_{1850}}$	ρ_1850_ = 1,850 ρ_1430_ = 1,432	Yu et al., 2000

**TABLE 2:** List of indices developed in this study.

Index	Formula	Wavelengths (nm)
Water Content Index (WCI)	$\mathrm{WCI}=\frac{\left(\rho_{686}-\rho_{955}\right)}{\left(\rho_{955}-\rho_{548}\right)}$	ρ_686_ = 686 ρ_955_ = 955 ρ_548_ = 548
Water Potential Index 1 (WPI1)	$\text{WP}1=\frac{\left(\rho_{665}-\rho_{715}\right)}{\rho_{715}}$	ρ_665_ = 660–670 ρ_715_ = 710–720
Water Potential Index 2 (WPI2)	$\text{WP}2=\frac{\left(\rho_{665}+\rho_{1457}\right)}{\left(\rho_{715}+\rho_{1457}\right)}$	ρ_665_ = 660–670 ρ_715_ = 710–720 ρ_1457_ = 1,457
Adjusted Red Green ratio Index (ARGI)	$\mathrm{ARGI}=\frac{\left(2*\rho_{650}\right)}{\left(\rho_{551}-\rho_{639}\right)}$	ρ_650_ = 600–700 ρ_551_ = 551 ρ_639_ = 639
Inverse Normalized Ratio index (IND_715/655_)	${\text{IND}}_{715/655}=\frac{\left(\rho_{715}+\rho_{655}\right)}{\left(\rho_{715}-\rho_{655}\right)}$	ρ_715_ = 710–720 ρ_655_ = 640–670
Ratio index 953/520 (R_953/520_)	${\text{R}}_{953/520}=\frac{\rho_{953}}{\rho_{520}}$	ρ_953_ = 953 ρ_520_ = 520
Ratio index 960/699 (R_960/699_)	${\text{R}}_{960/699}=\frac{\rho_{960}}{\rho_{699}}$	ρ_960_ = 960 ρ_699_ = 699
Ratio index 953/492 (R_953/492_)	${\text{R}}_{953/492}=\frac{\rho_{953}}{\rho_{492}}$	ρ_953_ = 953 ρ_520_ = 492
Normalized Difference index 1407/1862 (NDI_1407/1862_)	${\text{NDI}}_{1407/1862}=\frac{\rho_{1407}-\rho_{1862}}{\rho_{1407}+\rho_{1862}}$	ρ_1407_ = 1,407 ρ_1862_ = 1,862
Ratio 1451/1263 (R_1451/1263_)	${\text{R}}_{1451/1263}=\frac{\rho_{1451}}{\rho_{1263}}$	ρ_1451_ = 1,451 ρ_1263_ = 1,263

### Model Development

Physiological trait predictions were accomplished with index-based models and PLSR models. The aim of this modeling approach was to develop prediction models that can predict both developmental, diurnal and drought-induced changes in physiological traits during the acute drought period. This was achieved by training the models with physiological measurements and images collected at different time points during the day and on multiple days during the experiment. An 80% portion of the dataset (142 images of gas exchange- and fluorescence-measured plants and 88 images of Ψ- and WC-measured plants) was used to train the models, while 20% (36 and 23 images of gasexchange/fluorescence- and Ψ/WC-measured plants, respectively) was set aside for validation. The physiological traits that were selected for modeling consisted of A, E, gs, ΦCO2, ΦPS2, Fv′/Fm′, Ψ, and WC. The index-based models had a physiological trait as dependent and an index as independent variable. They were created with the “lm” function of the “stats” R package.

The PLSR models were developed with the PLSR function (“pls” R package) using the classical orthogonal score algorithm and Leave One Out Cross-Validation (Mevik et al., 2016). This technique has been widely used to construct predictive models of data that have more variables than observations and are highly collinear, such as hyperspectral data (Geladi and Kowalski, 1986; Pandey et al., 2017; Ge et al., 2019). The PLSR algorithm will reduce the dimensionality of the data, by extracting latent variables or components that account for most of the co-variance between the dependent and predictor variables in the data, while accurately modeling the trait of interest. The number of latent variables the model used will strongly influence the accuracy and overfitting of the model. To optimize the tradeoff between accuracy and overfitting, the predicted sum of squares was used to select the number of components (Chen et al., 2004). This was accomplished by determining the number of components at which the Root-Mean-Square Error (RMSE) of the predicted sum of squares was minimal (Wold et al., 2001; Yendrek et al., 2017). The contribution of each wavelength to the PLSR model was determined using the variable importance in the projection (VIP), which reflects the importance of each wavelength in the model (Chong and Jun, 2005). The VIP was used to perform feature selection using the procedure of Korn et al. (2010). The accuracy of the index-based and PLSR models were evaluated by calculating the RMSE and R-squared (*R*^2^) of the Leave One Out Cross-Validation and test data predictions with the postResample function of the “caret” R package (Kuhn et al., 2018).

### Statistics

The strong correlation between adjacent wavelengths produced multicollinearity. Wavelengths were therefore subdivided into groups based on a correlation threshold of 0.80. This resulted in 11 groups for which representative wavelengths were selected, namely 523; 551; 658; 708; 721; 976; 1,482; 1,694; 1,937; 2,110; and 2,321 nm (Supplementary Table 1). These wavelengths were used to evaluate the effect of drought treatments on the whole plant and light class population averages for each time point by performing a mixed model analysis. The same modeling approach was used to test the effect of drought on the non-destructive physiological traits, while a linear model was applied for the destructive measurements. To find associations between the physiological traits and reflectance a mixed and linear model was used for the non-destructive and destructive measurements, respectively. Time of imaging effects on the EXP reflectance data was investigated with a linear model created for the sixth day after the drought initiation. To incorporate the time of day effect in the drought effect analysis of the intermediate light class the data was reanalyzed with a factorial ANOVA that tested treatment differences within each hour for each day. This ANOVA analysis was also used to evaluate the effect of drought on indices. A *P*-value correction was performed during every statistical analysis. More details on the models and the type of *P*-value corrections can be found in the Supplementary Materials and Methods 2.

## Results

### Spectral Data Acquired in a Proximal Imaging Setup Are Strongly Influenced by Variations in Illumination

**FIGURE 1:**
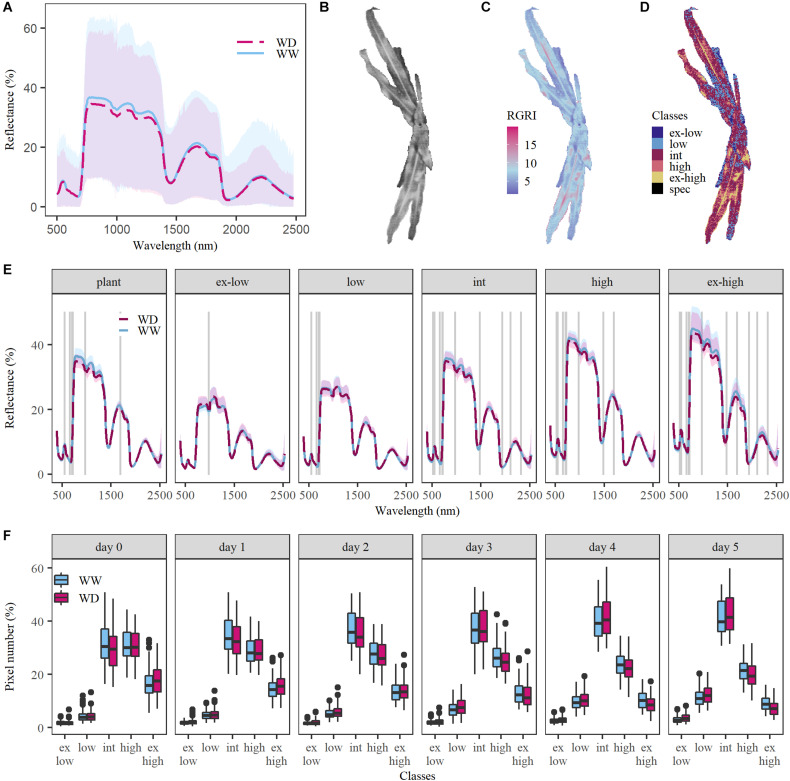
Confounding effects of within-plant illumination variation on the ability to detect drought in the VNIR and SWIR spectra. Data of the sixth day after the onset of drought was used to visualize the illumination effects. **(A)** Within-plant variation in relative reflectance (in %) for one well-watered (WW, light blue) plant and one plant grown under water deficit conditions (WD, red) with a pixel number of 9,144 and 5,743, respectively. The blue and red dashed lines represent the average reflectance, while the blue and pink shaded areas show the relative reflectance range within the WW and WD plants. **(B)** Within-plant variation in brightness. The brightness of each plant pixel was obtained based on relative reflectance in the red (664 nm), green (539 nm) and blue (429 nm) wavelength bands. **(C)** Within-plant variation of the Red Green Ratio Index (RGRI). **(D)** Distribution of the light classes within one plant. Six light classes were created: extremely low (ex-low), low, intermediate (int), high, extremely high (ex-high) light and specular reflection (spec). The latter was excluded from the analysis. **(E)** Average reflectance (in %) for WW and WD plants for the whole plant (left) and the five light classes (left to right). The gray lines indicate at which representative wavelengths (523; 551; 658; 708; 721; 976; 1,482; 1,694; 1,937; 2,110; and 2,321 nm) drought had a significant effect on relative reflectance (*P* < 0.05). **(F)**, Boxplots of the percentage of pixels in each light class for five subsequent days of the drought period after its initiation on day 0. The horizontal line within the box represents the median of 121–146 plants, while the lower and upper ends of the box indicate the first and third quartile. The lines below and above the box represent the minimum and maximum values and the outliers are marked with a black dot.

The reflectance data collected in our proximal imaging setup showed a high within-plant variability (Figure 1A), which was attributed to the variation in illuminance (total light flux per unit area) across the plant surface and the amount of reflected light detected by the camera sensors. Two factors determine the illuminance: the intensity of the light source and the plant surface geometry (Sandmeier et al., 1998; Behmann et al., 2015). Due to the high resolution of proximal hyperspectral imaging, the influence of geometry was very prominent as can be seen in the top-view brightness image of a maize plant (Figure 1B), where the distichous leaf organization causes brightness variations between and within leaves. These variations were attributed to differences in the distance between the leaf and the light source, shading, and the angle at which the light reached the leaf surface (angle of incidence). This had severe consequences for the interpretation of the hyperspectral data. The overall high within-plant variability in RGRI values (Figure 1C), which is a popular index to monitor the anthocyanin–chlorophyll ratio, corresponded with the observed high variation in brightness (Figure 1B), suggesting that variation resulted from differences in light exposure, and showing that it can mask potential effects of drought on reflectance.

Several methods have been proposed to remove the geometry-induced reflectance variation (Mishra et al., 2020a), such as hyperspectral 3D models (Liang et al., 2013; Behmann et al., 2016), standard normal variate (SNV) transformation (Vigneau et al., 2011; Asaari et al., 2018), variable sorting for normalization (Mishra et al., 2020b), light classification or calculating plant averages. 3D models and SNV transformation have been shown to remove linear-illumination effects, while light classification and plant averaging are also able to reduce variation caused by non-linear shading effects in thermal images (Leinonen and Jones, 2004; Jerbi et al., 2015).

In this study, two methods to reduce the within-plant variability were compared: the averaging of plant spectra, and classification based on brightness as a proxy for illumination. Brightness was subdivided into five light classes by performing an unsupervised k-means classification on a training dataset containing the different treatments and plant developmental stages. Thresholds for brightness were based on the classification results and applied to the whole dataset. Pixels that fell beyond the uppermost threshold were classified as specular reflection and removed from the analysis. To reduce the dimensionality of the data, an average plant spectrum was calculated for each of the five light classes: extremely low, low, intermediate, high and extremely high light. They showed up to 2.3 ± 0.4% difference in relative green reflectance between subsequent classes, which was related to their pixel composition. The extremely low and low light classes contained mainly shaded leaf pixels, whereas the higher light classes consisted of illuminated plant pixels (Figure 1D). Edge pixels were observed in both extremely low and extremely high light classes, while vein pixels were mainly present in the extremely high light class. To investigate how the pixel composition affected drought detectability, 11 wavelengths were selected (523; 551; 658; 708; 721; 976; 1,482; 1,694; 1,937; 2,110; and 2,321 nm) and the effect of drought was evaluated for each light class-wavelength combination. This analysis showed that classes containing shaded leaf parts (extremely low and low) received and reflected less light, resulting in less significant differences in relative reflectance between treatments (1–4 wavelengths, *P* < 0.05) compared to the higher light classes (8–11 wavelengths, *P* < 0.01, Figure 1E). The quantity of shaded and illuminated plant parts changed during the V5–V7 drought period (Figure 1F, day 0–5). The high and extremely high light pixel number decreased, as the plants developed more leaves, while the number of low and intermediate light pixels increased. The intermediate light class had the highest relative pixel number during the whole experiment and was consequently the most representative class (Figure 1F). Also, shaded and vein pixels were absent and therefore the intermediate light class was selected for the comparison with the overall average plant spectrum as an alternative data processing method. Light classification and plant averaging were compared for their ability to detect drought effects on the sixth day of the soil dehydration period, when these were maximal. A more pronounced drought effect was obtained in the intermediate light class than in the plant average spectra, with 10 and 6 significantly affected wavelengths, respectively (*P* < 0.05) (Figure 1E). Due to the clear difference in methods, it was decided to use the intermediate light class for this case study.

### Proximal Hyperspectral Imaging Systems Can Detect Subtle Diurnal Changes in Plant Physiology

**FIGURE 2:**
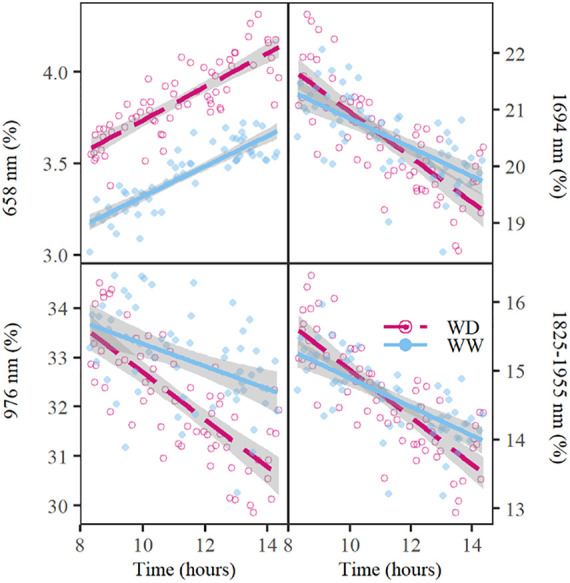
Diurnal changes in relative reflectance at 658; 976; and 1,694 nm and the water absorption trough with the ridge at 1,825 nm and the valley at 1,955 nm on day 6 of the drought period. The well-watered (WW) and water deficit (WD) treatments are indicated with a blue line or dot and a red dashed line or circle, respectively. The lines show the average trend of the treatment, whereas the dots and circles represent the relative reflectance of individual plants at the respective wavelengths. The gray shading around the lines indicate the standard error of relative reflectance. The water absorption trough depth values were calculated as the difference in relative reflectance between 1,825 and 1,955 nm.

The reduction of the illumination effects mainly improved the ability to detect drought in the visible, red-edge and SWIR regions of the electromagnetic spectrum, whereas the NIR region still showed a high variability (Figure 1E). The time of imaging of individual plants, which ranged from 8 AM to 3 PM on day 6 in Figure 1E, affected NIR reflection, because significant decreases in NIR (976 nm), red-edge (708 and 721 nm) and SWIR (1,482; 1,694; 1,937; 2,110; and 2,321 nm) relative reflectance (*P* < 0.001) was observed over this timespan (Tables 3, 4 and Figure 2). The time-of-day effect was also found in the visible region (523, 551, and 658 nm), where an increase in relative reflectance was observed as the day progressed (*P* < 0.001) (Table 3, Figure 2 and Supplementary Figure 1). These time-of-day effects were observed in both WW and WD treatments and differed significantly between treatments for red-edge (721 nm), NIR (976 nm), and one SWIR wavelength (1,694 nm). The WD treatment showed a stronger negative slope than the WW treatment, which resulted in a more pronounced drought effect in the afternoon (*P* < 0.05) (Tables 3, 4 and Figure 2). Diurnal changes were not limited to specific wavelengths but could also be observed in the depth of the troughs, which correspond to regions where reflectance decreases abruptly. Of the five troughs in the VNIR and SWIR regions with their dips at 979; 1,232; 1,445; 1,825; and 1,955 nm (Figure 1E), three (979; 1,445; and 1,955 nm) showed a significant negative diurnal trend for both WW and WD plants (Figure 2 and Supplementary Figure 1). A treatment difference was only observed in the 1,955 nm trough (*P* < 0.01) (Table 5 and Figure 2).

**TABLE 3:** Slopes of the time-of-day effects on VNIR relative reflectance for the sixth day after the onset of drought.

Wavelength	Treat	Slope	Significance slope	Significance treatment difference	Significance wavelength differences
523 nm	WW	0.0857	***		all wavelengths (***) except 658 nm
	WD	0.0911	***		
551 nm	WW	0.0303	**		all wavelengths (**)
	WD	0.0035			
658 nm	WW	0.0828	***		all wavelengths (***) except 523 nm
	WD	0.0916	***		
708 nm	WW	–0.0690	***		all wavelengths (*) except 1,482; 1,937; 2,110; and 2,321 nm
	WD	–0.0774	***		all wavelengths (**) except 1,937 nm
721 nm	WW	–0.2087	***	**	all wavelengths (**) except 976 and 1,694 nm
	WD	–0.3262	***		all wavelengths (*) except 1,694 nm
976 nm	WW	–0.2281	**	**	all wavelengths (*) except 721; 1,482; 1,694; 2,110; and 2,321 nm
	WD	–0.4790	***		all wavelengths (*) except 1,694 nm

Slopes are compared between treatments and between wavelengths or troughs. *P < 0.05. **P < 0.01. ***P < 0.001

**TABLE 4:** lopes of the time-of-day effects on SWIR relative reflectance for the sixth day after the onset of drought.

Wavelength	Treat	Slope	Significance slope	Significance treatment difference	Significance wavelength differences
1,482 nm	WW	–0.0972	***		All wavelengths (*) except 708; 976; 2,110; and 2,321 nm
	WD	–0.1600	***		All wavelengths (**) except 2,110 and 2,321 nm
1,694 nm	WW	–0.2546	***	*	All wavelengths (***) except 721 and 976 nm
	WD	–0.4002	***		
1,937 nm	WW	–0.0363	**		All wavelengths (*) except 708 nm
	WD	–0.0425	***		
2,110 nm	WW	–0.0999	***		All wavelengths (**) except 708; 976; 1,482; and 2,321 nm
	WD	–0.1607	***		All wavelengths (***) except 1,482 and 2,321 nm
2,321 nm	WW	–0.1108	***		All wavelengths (*) except 708; 976; 1,482; and 2,110 nm
	WD	–0.1747	***		All wavelengths (**) except 1,482 and 2,110 nm

Slopes are compared between treatments and between wavelengths or troughs. *P < 0.05. **P < 0.01. ***P < 0.001.

**TABLE 5:** Slopes of the time-of-day effects on absorption troughs for the sixth day after the onset of drought.

Trough	Treat	Slope	Significance slope	Significance treatment difference	Significance trough differences
979	WW	–0.1961	***		1,445 and 1,825 (***)
	WD	–0.2135	***		All troughs (*)
1,232	WW	–0.0365	*		All troughs(*)
	WD	–0.0290			979; 1,445; and 1,955 (***)
1,445	WW	–0.2280	***		1,232 and 1,955 (***)
	WD	–0.3425	***		979; 1,232; and 1,955 (*)
1,825	WW	0.0021			All troughs (*)
	WD	–0.0013			979; 1,445; and 1,955 (***)
1,955	WW	-0.2086	***	**	1,232 and 1,825 (***)
	WD	–0.3442	***		979; 1,232 and 1,825 (***)

The trough depths are estimated by taking the difference in relative reflectance between the highest (852; 1,100; 1,232; 1,650; and 1,825 nm) and the lowest (979; 1,232; 1,445; 1,825; and 1,955 nm) wavelengths of the troughs. Slopes are compared between treatments and between wavelengths or troughs. *P < 0.05. **P < 0.01. ***P < 0.001.

**FIGURE 3:**
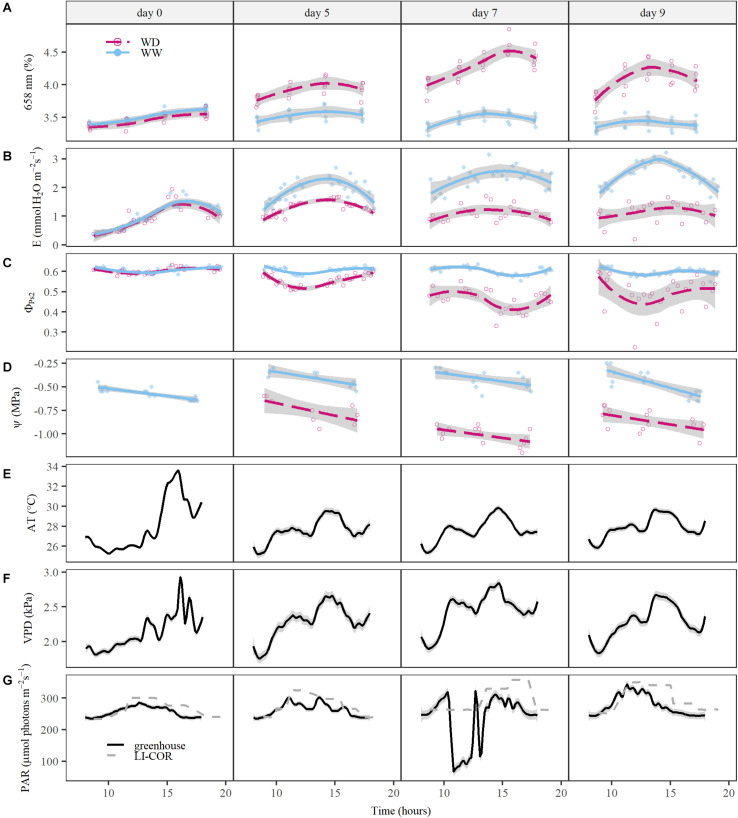
The responses of relative reflectance at 658 nm and physiological traits to diurnal variations in environmental conditions and drought. Relative reflectance and physiological trait measurements of well-watered (WW) and water deficit (WD) treatment plants are compared during the drought period (day 0, 5, 7 and 9). The average trends of the WW and WD treatments are indicated by a blue line and red dashed line, respectively. The individual measurements of the treatments are visualized with a blue dot (WW) or red circle (WD). **(A)** Relative reflectance at 658 nm. **(B)** Transpiration rate (E, mmol H_2_O m^–2^s^–1^). **(C)** Effective quantum yield of photosystem 2 (Φ_*PS*2_). **(D)** Leaf water potential (Ψ, MPa). **(E)** Average greenhouse air temperature (AT, °C). **(F)** Vapor pressure deficit (VPD, kPa). **(G)** Photosynthetically active radiation (PAR, μmol photons m^–2^s^–1^). In panels **(E–G)**, the black line represents the greenhouse environmental conditions, which is supplemented in panel **(G)** with the PAR settings of the LI-COR LI-6400 (gray dashed line). The gray shading around the lines indicate the standard error of relative reflectance, physiological traits and environmental conditions.

The observed diurnal variation had major implications for spectrum interpretation and coincided with physiological trait changes and variations in environmental conditions (Figure 3). Φ_*PS*2_ showed a minimum around noon corresponding to the PAR maximum in the greenhouse (Figures 3C,G). Leaf Ψ decreased during the day with the lowest values in the afternoon/evening, when E and VPD had passed their peak (Figures 3B,D,F). Significant strong correlations with VPD were observed in plant reflectance at 721 nm (correlation coefficient r, r_WW_ = –0.80, r_WD_ = –0.79, *P* < 0.05), 658 nm (r_WW_ = 0.74, r_WD_ = 0.77, *P* < 0.05), 523 nm (r_WW_ = 0.74, r_WD_ = 0.67, *P* < 0.05), and the 979 nm trough (r_WW_ = –0.80, r_WD_ = –0.78, *P* < 0.05). These wavelengths were in turn significantly correlated with E and Ψ. The reflectance at 658 nm showed a similar diurnal pattern as E with a maximum in the afternoon (Figure 3A), while 721 nm was negatively correlated with E. The correlation between E and reflectance at 658 nm was significant in both WW (r_WW__658 = 0.65, P < 0.05) and WD (r_WD__658 = 0.48, *P* < 0.05) treatments on all sampling days of the experiment, except for the WD treatment on day 7. This was the last day of the acute drought period, when E was at its minimum and 658 nm reflectance at its maximum. On this day, a stronger diurnal effect on reflectance than on E was observed, suggesting an additional effect of drought on reflectance at 658 nm not directly related to E. A positive relationship was found between Ψ and the 979 nm trough, but was only significant for the WW treatment (r_WW_ = 0.56, P < 0.05). The correlation with 523 and 658 nm was significantly negative for both treatments (523 nm: r_WW_ = –0.51, r_WD_ = –0.46, *P* < 0.05; 658 nm: r_WW_ = –0.67, r_WD_ = –0.41, *P* < 0.05).

### Drought-Induced Reflectance Changes Are Correlated With Physiology

Withholding water from maize plants affected leaf reflectance in both the VNIR and SWIR areas of the electromagnetic spectrum. An increase in reflectance was observed in the blue-green, red, and SWIR region (523; 658; 1,482; and 2,110 nm), while a decrease was present in the green, red-edge and NIR region (551, 708, 721, and 976 nm) (*P* < 0.01, Supplementary Figures 3,4). Red reflection was the most sensitive to drought, as significant treatment differences were observed already two days after the onset of drought (*P* < 0.01, Supplementary Figure 3). Reflection in the blue-green, red-edge and SWIR (1,482 nm) region showed drought effects on the fourth day (*P* < 0.01, Supplementary Figures 3,4). NIR reflectance was the least sensitive to acute drought as it only showed significant differences between treatments near the end of the drought period (day 6, *P* < 0.01, Supplementary Figure 3). The ability to detect these drought effects was influenced by the interaction between time-of-day and treatment, as most treatment effects were first detected in the early afternoon. This interaction was especially pronounced for the red-edge (721 nm) and NIR (976 nm) wavelengths, which have been positively correlated with leaf thickness and negatively correlated with the WC (Slaton et al., 2001; Neilson et al., 2015). During the morning, E increased (Figure 3B), resulting in a decrease in WC, Ψ (Figure 3D and Supplementary Figure 2H) and leaf thickness (Syvertsen and Levy, 1982; Mcburney, 1992; Jinwen et al., 2009). This decrease in leaf thickness was probably more pronounced in WD than WW plants, translating into a stronger drop in WD plants’ NIR and red-edge reflection, and therefore a larger treatment difference in the afternoon.

**FIGURE 4:**
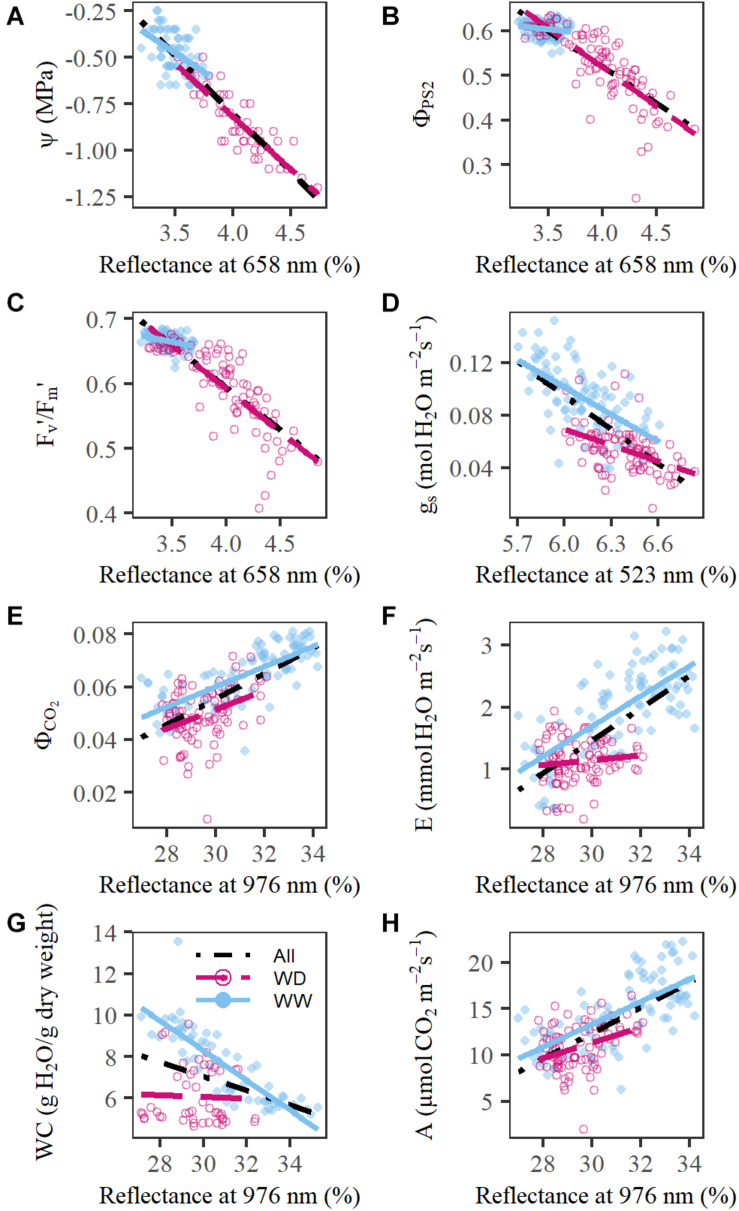
Relationship between relative reflectance and physiological traits. **(A)** red (658 nm) vs. leaf Ψ (MPa). **(B)** red (658 nm) vs. Φ_*PS*2_. **(C)** red (658 nm) vs. F_*v*_′/F_*m*_′. **(D)** Blue-green (523 nm) vs. g_*s*_ (mol H_2_O m^–2^s^–1^). **(E)** NIR (976 nm) vs. Φ_*CO*2_. **(F)** NIR (976 nm) vs. E (mmol H_2_O m^–2^s^–1^). **(G)** NIR (976 nm) vs. leaf WC (g H_2_O/g dry weight). **(H)** NIR (976 nm) vs. A (μmol CO_2_ m^–2^ s^–1^). The black dot-dashed line indicates the relationship without treatment effect, whereas the blue line and the red dashed line represent the relationship for the well-watered (WW) and water deficit (WD) treatment, respectively. Measurements on individual plants are indicated by blue dots (WW) and red circles (WD).

Drought had a negative effect on the measured physiological traits, which was observed earlier for E, g_*s*_, F_*v*_′/F_*m*_′, WC, and Ψ and later for A, Φ_*PS*2_, and Φ_*CO*2_ (*P* < 0.05, Figure 3 and Supplementary Figure 2). All wavelength groups, except for the 2,321 nm group, correlated significantly with one or more of these traits (Figure 4 and Supplementary Table 2). The most promising reflectance–physiology relationships were observed between red reflectance (658 nm) and Ψ, Φ_*PS*2_ or F_*v*_′/F_*m*_′ with negative slopes (*P* < 0.0001, Figures 4A–C). The relationships with red reflection were significantly different between WW and WD treatments (two-tailed Student’s *t*-test, *P* < 0.0001), except for the relation with Ψ. This suggests that the underlying relationship is the same for diurnal, developmental and drought-induced changes. Other treatment-independent, positive relationships were observed between 976 nm and Φ_*CO*2_ or A (*P* < 0.001, Figures 4E,H). Significant treatment effects were present in the relationships of 523 nm with g_*s*_, 976 nm with WC and were very pronounced for 976 nm and E, as correlations were only present in the WW treatment (Figures 4D–G, *P* < 0.001). The lack of correlation in the WD treatment may be related to the limited range of E values resulting from increased stomatal closure. The drought effects on physiology were not limited to direct effects but were also influenced by differences in development, which are discussed in the Supplementary Results 1.

### Both Index-Based and PLSR Models Can Accurately Predict Physiological Traits From Hyperspectral Data

The significant relationships between reflectance and physiology can be used for automated plant phenotyping by calculating vegetation indices for physiological traits or by creating PLSR-based physiological trait prediction models. Here, 10 publicly available and 10 new indices (Tables 1, 2) were evaluated for their ability to detect drought and their prediction accuracy of physiological traits by creating index-based linear models. The accuracy of these models was subsequently compared to PLSR models of the same traits, to evaluate which of the two methods was more suitable to monitor drought effects. The physiological traits that were selected for this comparison were A, E, g_*s*_, Φ_*PS*2_, F_*v*_′/F_*m*_′, Ψ, and WC, as these were significantly affected by the drought treatment. The published indices under evaluation included WC indices (Water Band Index, Moisture Stress Index and Relative Water Content index), photosynthetic efficiency indices (PRI and RGRI), pigment content indices (Modified Chlorophyll Absorption Ratio Index and Carotenoid Reflectance Index 1) and indices that used red or NIR reflection (NDVI, Ratio Vegetation Index 870/610, Ratio NIR/510) (for references see Table 1). Large differences in drought sensitivity were observed in both the existing and new indices, ranging from early drought responses to no consistent effects. The indices were grouped accordingly: very sensitive, sensitive, moderately sensitive and insensitive. The results are presented in Supplementary Results 2.

**FIGURE 5:**
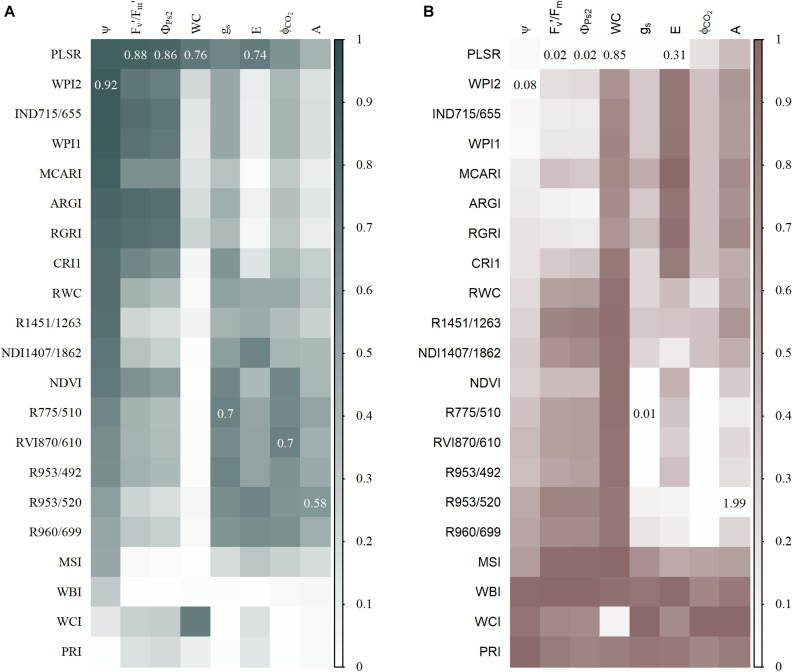
Test prediction accuracy of partial least square regression (PLSR) and index-based models. **(A)** A heatmap of *R*^2^ values, with higher values plotted in a gray color and lower values in white. The highest *R*^2^ value for each physiological trait is shown in the representative square. **(B)** A heatmap of scaled RMSE in which lower values are indicated with a white color and higher values with brown. The lowest unscaled RMSE values are shown in the representative squares. The PLSR models of F_*v*_′/F_*m*_′, Φ_*PS*2_, g_*s*_, Φ_*CO*2_, and A used seven components, models of WC and E used 10 components, and the model of Ψ eight components.

Partial least square regression has been commonly used to estimate physiological traits from hyperspectral data. It has the advantage of using all the available wavelengths, but some may turn out to add additional noise. Here, the use of PLSR slightly improved the test RMSE of E, Φ_*PS*2_, WC, and F_*v*_′/F_*m*_′ by 7, 13, 8, and 12%, respectively (Figure 5). However, Ψ, g_*s*_, Φ_*CO*2_, and A showed a lower PLSR accuracy than the index-based models with a reduction in the RMSE of 10, 5, 17, and 24%, respectively (Figure 5). Maize reflectance showed a weaker relationship with A compared to the other physiological traits, resulting in a low accuracy of both index-based and PLSR models. The importance of each wavelength in the PLSR models was determined by calculating the VIP (Chong and Jun, 2005). The 30 wavelengths with the highest VIP scores were compared to those used by the indices (Supplementary Table 3), showing that most PLSR models contained wavelength regions similar to those important in the index-based linear models. The F_*v*_′/F_*m*_′, Φ_*PS*2_, and Ψ models consistently used red and/or red-edge reflectance, while for the predictions of Φ_*CO*2_ and g_*s*_, NIR reflectance was always important. The water absorption regions were also conserved between the index-based and PLSR models of E, A, and WC. The wavelengths that were important in PLSR can be found in Supplementary Table 3. One wavelength region around 2,500 nm was never used in the index-based models, but had the highest VIP value and was very important for the PLSR models, although it was located at the edge of the SWIR spectrum and may have been noisy. The comparison of PLSR and index-based models showed that when a strong relationship between reflectance and the physiological trait is present, both PLSR and index-based models can accurately estimate physiological traits from hyperspectral data.

## Discussion

### Within-Plant Illumination Differences Cause Large Non-biological Variation in Reflection

In this study of proximal hyperspectral imaging in the context of an automated phenotyping platform, large non-biological variation in reflectance caused by illumination differences due to plant geometry masked subtle biological differences in reflectance, including diurnal patterns and drought responses. To manage the illumination effects, the use of artificial diffuse light has been proposed to reduce the effects of geometry on hyperspectral data, but it does not remove differences caused by the distance between the leaves and the light source (Thomas et al., 2018). These effects can be reduced by combining hyperspectral data with high-resolution 3D plant models (Behmann et al., 2016), created by means of stereo or rotating red-green-blue and hyperspectral camera systems that image the plants from different angles (Liang et al., 2013). Once 3D information of each hyperspectral pixel is available, the angle and the distance between the pixel and the light source can be calculated, and their effect can be removed by a correction factor for each pixel determined from the light field of illumination intensities (Behmann et al., 2016). Distance and inclination effects can also be removed by using the 3D information to estimate the parameters of a linear reflectance model (Vigneau et al., 2011; Asaari et al., 2018), by incorporating distance and angle information in trait prediction models (Roscher et al., 2016) or by developing a bidirectional reflectance distribution function (Behmann et al., 2016). Combining 3D information with hyperspectral data is a promising approach to reduce the illumination effects, however, this information is not always available, and the sheer volume of this extra data further challenges storage and processing capacities. Consequently, several alternative methods have been proposed, the simplest of which is to reduce the within-plant variation by calculating plant averages. This method removes all spatial information and reduces spectral variation without taking the source of the variation into account. By ignoring the illumination effects (variation source) a positive relationship between reflectance and the distance between the plant and the light source is created (Asaari et al., 2018), which complicates hyperspectral interpretation when plants differ in height. Vigneau et al. (2011) and Asaari et al. (2018) have proposed to model a correction using a linear reflection model, which assumes a multiplicative effect of distance and inclination and an additive effect of specular reflection. Alternative approaches to remove linear effects are spectra normalization methods such asthe SNV transformation and variable sorting normalization (Vigneau et al., 2011; Asaari et al., 2018; Mishra et al., 2020b). The SNV transformation can cause a wavelength shift in the treatment differences (Fearn, 2009), which prevents the use of existing indices and complicates the biological interpretation of the spectra. More details on the available linear illumination correction approaches have been described in the review paper of Mishra et al. (2020a).

The methods discussed above have focused on linear effects such as distance and inclination, whereas non-linear effects such as shading also strongly influence plant reflectance. Illumination classification, which subdivides plant parts based on the amount of illumination, can reduce the reflectance variation caused by linear and non-linear effects as both these factors affect brightness. This method has been used to separate sunlit and shaded leaf pixels in both hyperspectral remote sensing and thermal images (Jerbi et al., 2015). Plant pixels can be subdivided by splitting the leaves into segments from the base to the tip (Tirado et al., 2020) or by performing a brightness-based classification. In this study, the latter was evaluated by subdividing plant pixels into five illumination classes. A k-means clustering approach was used as a guide to determine the brightness thresholds, which subdivided plants in the minimum number of clusters required to reduce the illumination effects. The clustering approach was applied in a less conventional way without using the optimal number of clusters, as this amount varied strongly between optimization methods resulting in too little (1 cluster) or too many clusters (Charrad et al., 2014). The brightness-based classification reduced the within-plant variance in reflectance by 47 ± 24% and improved the detectability of drought effects especially in the classes that contained sunlit plant pixels (intermediate and high light). However, by using only a subset of the plant pixels, the method is less suitable for studies that focus on the spatial distribution of traits within the plant. In these situations the use of the above described linear correction methods is more appropriate. The main advantages of the illumination classification compared to the use of 3D information and normalization is that it reduces both linear and non-linear illumination effects, it does not require additional information on plant geometry and it allows the use of published indices, as the data is not transformed. The combination of these advantages makes this method more intuitive and accessible for plant scientists. The brightness-based clustering, including the appropriate number of clusters, the brightness thresholds and the optimal cluster for analysis, is setup-specific and requires a preliminary analysis as described in this study.

### Hyperspectral Data Allow the Detection of Diurnal and Drought-Induced Changes in Physiology

By reducing the illumination-induced variation, subtle diurnal trends in plant reflectance were detected, which were correlated with changes in plant physiology. Diurnal effects are unavoidable in the PHENOVISION platform, because a 6-h imaging cycle is required for almost 400 individual plants. On the other hand, the results show that hyperspectral imaging delivers high-resolution plant physiology-related data and that the current setup allows the monitoring of diurnal changes in plant physiology, which is important in the timely detection of and genotypic specific sensitivity to stress, and because treatment effects can depend on the time of sampling or imaging (Figure 2; Žibrat et al., 2019). Consideration of the time-of-day has mainly been limited to the PRI index created to detect diurnal changes in the epoxidation state of the xanthophyll cycle pigments and in the photosynthetic efficiency (Gamon et al., 1992). Other studies have shown a significant correlation between PRI and F_*v*_′/F_*m*_′ on both diurnal and seasonal scales (Garbulsky et al., 2011; Zhang et al., 2016). Here, diurnal trends in reflectance were observed in a wide spectral range, including blue-green (521 nm), red (658 nm), red-edge (708 nm), NIR (721 nm), and SWIR (1,482; 1,694; 1,937; 2,110; and 2,321 nm) wavelengths. Diurnal trends in red and NIR reflection have been observed before, but these effects were attributed to the influence of soil reflection, differences in solar angle and plant geometry (Asrar et al., 1985; Gamon et al., 1992). In PHENOVISION, these factors do not play a major role. Instead, the diurnal trends showed correlations with VPD, E, and Ψ. The strongest relationships were observed with reflection of red, NIR and the water absorption trough at 979 nm. NIR and water absorption trough reflectance have been related to WC, leaf thickness and anatomy (Slaton et al., 2001; Neilson et al., 2015), which can change during the day as the plant loses water through E (Syvertsen and Levy, 1982; Mcburney, 1992; Jinwen et al., 2009). These changes may thus explain the negative correlation of NIR and the 979-nm trough with E and VPD. A biological explanation for the strong relationship between red reflectance and E, Ψ, or VPD is less straightforward, as no clear diurnal trends in pigment content were observed. Red absorption and reflectance can be affected by the amount of light scattering within the tissue by changing cell size and shape or by changing the positioning of organelles, such as chloroplasts. Changes in leaf thickness and water loss can affect mesophyll density and cell size and shape (Chartzoulakis et al., 2002; Scippa et al., 2004; Sancho-Knapik et al., 2011; Wuyts et al., 2012), however, the few studies that investigated these effects focused on different degrees of drought to reduce leaf WC instead of looking at diurnal variations. Diurnal changes in chloroplast positioning have been observed in C4 plants as a light avoidance response with aggregative movements in the afternoon to increase mutual shading of chloroplasts (Yamada et al., 2009). Maai et al. (2011) showed that these light avoidance and aggregative movements are triggered by high levels of blue light and abscisic acid (ABA). A strong correlation was observed between red reflection and VPD, which has been associated with ABA levels, an important phytohormone involved in the regulation of g_*s*_ (Merilo et al., 2018). g_*s*_ was monitored during the validation experiment but only a drought-induced decrease could be observed, suggesting that chloroplast movement mainly played an important role in the drought-induced increases in red reflection.

Drought effects on plant physiology and reflectance have been studied in both lab and field experiments. The relationship between plant reflectance and physiology is complex, because drought affects almost the whole measured plant spectrum, and physiological traits can influence multiple wavelengths directly or indirectly (Römer et al., 2012), which was confirmed here. The earliest effects were observed in the visible region, where an increase in red reflectance was present two days after the onset of the drought treatment. Increases in red reflectance attributed to changes in pigment content (Sims and Gamon, 2002; Sun et al., 2018) were not visible here, instead red reflectance showed a strong correlation with photosynthetic efficiency (Φ_*PS*2_ or F_*v*_′/F_*m*_′) and Ψ, suggesting that other factors, such as light scattering within the leaf and chloroplast positioning, may have impacted red reflectance during the drought treatment. The negative relationship between red reflectance and Ψ did not differ between treatments, implying that the underlying link is the same for developmental and drought-induced changes. Changes in light scattering will also affect NIR reflectance. The wavelength that represented NIR reflectance in this study (979 nm) is located in the NIR water absorption trough, which has been correlated with WC (Peñuelas et al., 1993; Corti et al., 2017). A correlation between WC and 976 nm was only present in the WW treatment, indicating that this wavelength could only detect diurnal and developmental changes in WC when there were no drought effects present (Figure 4), and that it is affected by multiple drought-induced physiological changes, of which WC is only a small part. Two additional water absorption troughs are present in the SWIR region (1,482 and 1,937 nm), which showed weak correlations with WC in both treatments. Correlations between these wavelengths and WC measurements have been observed in previous drought studies noting increases in SWIR reflectance as the drought effect progressed (Susič et al., 2018; Krishna et al., 2019). The strength of these relationships depended on the type of WC measurement, as stronger correlations were observed for the leaf WC per unit area than WC per leaf dry weight (Yu et al., 2000; Ceccato et al., 2001; Yi et al., 2013). A correction of the dry mass effect on SWIR reflectance is necessary to properly estimate the WC. This may be accomplished by combining SWIR with the NIR region that is only influenced by dry mass (740–900 nm) in indices or models.

### Predicting Physiological Traits Using Hyperspectral Index-Based Linear and PLSR Models

Hyperspectral imaging produces voluminous multidimensional data. One way to extract biological information is to reduce the dimensionality by calculating indices that only use a subset of the reflectance spectrum. A vast number of indices created for remote sensing applications are now used in proximal phenotyping studies. NDVI is a very popular index applied in several drought studies (Kim et al., 2011; Römer et al., 2012; Behmann et al., 2014; Sun et al., 2018). Its performance ranges from no drought-induced differences to a significant reduction in NDVI values. Behmann et al. (2014) observed that NDVI has a higher relevance in the senescence stages of barley, suggesting that this index is less suitable for early drought detection when drought does not affect pigment content and leaf senescence. This was confirmed here, because the NDVI only showed significant effects from day 4 of the drought treatment onwards, while the RGRI detected effects by the second day. In maize, drought treatments cause an increase in RGRI values (Sun et al., 2018), which can be attributed to changes in photosynthetic efficiency and/or pigment content. Because a mild drought was applied during these experiments, no clear differences in pigment content were observed, while photosynthetic efficiency (F_*v*_′/F_*m*_′ and Φ_*ps2*_) was significantly reduced and strongly correlated with the RGRI (Figure 5). The RGRI was also the only published index that correlated with one of the traits it was originally developed for. In addition, two WC indices (Relative Water Content index and R1451/1263) showed a relationship with Ψ and correlated with the plant water status. All existing WC indices were outperformed by the new water potential (WPI2) and water content (WCI) indices (*R*^2^ of 0.92 and 0.73, respectively). These indices combined water absorption wavelengths with red reflection, which was the most drought-sensitive wavelength. The poor performance of published WC indices has also been observed by Yi et al. (2013) and may be attributed to the influence of multiple structural and physiological traits that affect their transferability to other species, genotypes, developmental stages and experimental setups. In addition, many indices were created for remote sensing applications, where reflectance is affected by non-biological factors that do not influence reflectance in proximal sensing.

Calculating indices is the simplest method to analyze hyperspectral data, but it utilizes only a small subset of the available wavelengths with a potential loss of biological information. Multivariate techniques, such as PLSR, provide an alternative where the whole-plant spectrum is used by reducing the dimensionality using principal components. PLSR models have been created in several drought studies to predict water use traits, such as Ψ, WC, and g_*s*_ (Rapaport et al., 2015; Ge et al., 2016; Corti et al., 2017; Pandey et al., 2017; El-Hendawy et al., 2019). Three studies have compared the accuracy of index-based and PLSR models, demonstrating that these multivariate models performed equally or better than published indices (Hansen and Schjoerring, 2003; Atzberger et al., 2010; Heckmann et al., 2017; Yendrek et al., 2017; Ge et al., 2019). In this study, both slight accuracy improvements (7–13 %) and deteriorations (5–24 %) were observed when index-based models were compared to PLSR models. The similarity in the prediction accuracy of both methods can be explained by the fact that they use similar wavelength regions. The index-based or PLSR models with the highest prediction accuracy for the physiological traits Ψ, F_*v’*_/F_*m’*_, Φ_*PS*2_ (*R*^2^_*WPI2_index*_ = 0.92, *R*^2^_*PLSR*_ = 0.88, *R*^2^_*PLSR*_ = 0.86, respectively), incorporated red-edge and red reflection. These two wavelength regions were complemented by reflectance of the SWIR water absorption trough (1,457 nm) in the index-based Ψ model. The importance of the red-edge and the SWIR water absorption trough for Ψ predictions has been confirmed by the grapevine case study of Rapaport et al. (2015), which suggests that these wavelengths may be related to Ψ across different plant species. The importance of red-edge wavelengths in the prediction of drought-induced changes in physiology was not limited to Ψ, F_*v’*_/F_*m’*_, and Φ_*PS*2_, as this region received high VIP values in many physiological PLSR models, such as for g_*s*_, Φ_*CO*2_, and WC. High red-edge VIP values have also been observed in the WC PLSR models of Corti et al. (2017). Only the E PLSR model did not incorporate the red-edge in its top 30 wavelengths. To predict E, NIR, and SWIR reflectance plays a more prominent role. These wavelength regions have been related to WC and leaf internal structure, illustrating their importance in monitoring plant water use behavior. The usability of PLSR models for reviewing physiological traits was demonstrated in several studies, but the transferability of PLSR and index-based models to other studies or datasets is uncertain. Vigneau et al. (2011) observed that PLSR models were dataset-dependent and could not be applied on plants grown in a different environment. Fu et al. (2019) went further in showing that stacking regression models improved predictions of hyperspectral reflectance with photosynthetic capacities in field-grown tobacco over any individual model, including those based on PLSR. Obviously more robust models can be created in other ways such as using multiple genotypes or species, growing conditions, sensors and developmental stages.

## Conclusion and Future Perspectives

Hyperspectral imaging is now a demonstrated tool for plant phenotyping in automated platforms. In this case study, hyperspectral imaging was able to resolve diurnal changes in physiological traits, such as E, and detected the interaction between diurnal and drought-induced changes in plant physiology, which may mask drought effects. The imaging system was also able to satisfactorily monitor drought-induced changes in Ψ and photosynthetic efficiency with both index-based and PLSR models. The PLSR models performed similar or better than the index-based models for most of the physiological traits, as long as a decent correlation between physiology and reflectance was present. However, indices do not always require the development of models as they can also be used to investigate relative differences between treatments. The results observed in this study were only obtainable after correcting the additional illumination-induced reflectance variation, which was accomplished by performing a light classification. This pre-processing approach requires no additional information making it a more easily accessible method to remove illumination effects. Nevertheless, illumination is not the only factor; plant development, growing conditions, genotype and species will also influence reflectance by creating biological variation that may not have been accounted for during the establishment of the hyperspectral processing protocols. Development affected both reflectance and physiology in this case study, resulting in greater treatment effects across many wavelengths. These development effects will become more pronounced when plants are monitored during their whole development. To our current knowledge, no information of the effects of plant development on reflectance and its relation to physiology has been published. A better understanding of the influence of these different factors on reflectance and its relationship with physiology is crucial for the development of robust indices and multivariate models that can be used in plant phenotyping and screening of drought-tolerant varieties. The plant reflectance spectrum contains a vast amount of information about physiological and structural plant traits, such as leaf internal structure, leaf thickness, pigment and WC. Current knowledge about the causal relationships between reflectance and plant traits is limited and insufficient to explain the more complex interactions between reflectance and physiology that were observed in this case study. Several hypotheses were proposed to explain these relationships, but more research is needed to validate or refine these hypotheses.

## Data Availability Statement

The original contributions presented in the study are included in the article/Supplementary Material, further inquiries can be directed to the corresponding author/s.

## Author Contributions

NW, DI, HN, SMa, GB, SC-B, JV, and WB conceived the original screening and research plans. NW supervised the experiments. LV, NW, and SMe performed the experiments, collected hyperspectral data and physiological measurements. KD, KM, BC, and JD provided technical assistance during experiments. HS and SMe analyzed the hyperspectral data. SMe performed statistics and wrote the manuscript. NW, DI, and HN revised the manuscript. DI agreed to serve as the author responsible for contact and ensures communication. All authors contributed to the article and approved the submitted version.

## Conflict of Interest

GB was employed by company BASF Innovation Center Gent, Belgium. SC-B, JV, and WB were employed by BASF Corporation, USA. The remaining authors declare that this study received funding from BASF. The funder had the following involvement in the study: collaboratively conceived the original screening and research plans.

## Footnotes

1
https://www.psb.ugent.be/phenotyping/phenovision

## Supplementary Material

Supplementary Figure 1 Time-of-day effects on relative reflectance at 551, 708, 721, 1482, 1937, 2110 and 2321 nm, and the water absorption trough depths.

Supplementary Figure 2 The responses of physiological traits and indices to diurnal variations in environmental conditions and drought.

Supplementary Figure 3 Drought differences in VNIR reflectance.

Supplementary Figure 4 Drought differences in SWIR reflectance.

Supplementary Figure 5 The ability to detect drought effects using standard indices.

Supplementary Figure 6 The ability to detect drought of very sensitive and sensitive indices.

Supplementary Figure 7 The ability to detect drought effects of moderately sensitive and insensitive indices.

Supplementary Table 1 The representative wavelengths and the wavelength ranges that are most strongly correlated with them.

Supplementary Table 2 The relationship between relative reflectance and physiological traits.

Supplementary Table 3 Top 30 wavelengths with high VIP values for each of the PLSR models.

Supplementary Results 1 Maturation of maize plants affects physiology, reflectance and drought effects.

Supplementary Results 2 Drought sensitivity of new and existing indices.

Supplementary Materials and Methods 1 Pigment content extraction.

Supplementary Materials and Methods 2 Statistics.

## Abbreviations

3D: 3-dimensional
A: photosynthetic rate
E: transpiration rate
EXP: exploratory experiment
F_*v*_′/F_*m*_′: energy harvesting efficiency by oxidized PS2
g_*s*_: stomatal conductance of H_2_O
NDVI: normalized difference vegetation index
NIR: near-infrared
PAR: photosynthetically active radiation
PLSR: partial least square regression
PRI: photochemical reflectance index
R^2^: R-squared
RGRI: red green ratio index
RMSE: root-mean-square error
SNV: standard normal variate transformation
SWIR: shortwave-infrared
VAL: validation experiment
VIP: variable importance in the projection
VNIR: visible and near-infrared
VPD: vapor pressure deficit
WC: water content
WD: water deficit
WW: well-watered
Φ_*CO*2_: quantum yield based on CO_2_
Φ_*PS*2_: effective quantum yield
Ψ: water potential.

## References

[B1] Asaari M. S. M. Mishra P. Mertens S. Dhondt S. Inzé D. Wuyts N. (2018). Close-range hyperspectral image analysis for the early detection of stress responses in individual plants in a high-throughput phenotyping platform. *Isp. J. Photogram. Remote Sens.* 138 121–138. 10.1016/j.isprsjprs.2018.02.003

[B2] Asrar G. Kanemasu E. T. Yoshida M. (1985). Estimates of leaf area index from spectral reflectance of wheat under different cultural practices and solar angle. *Remote Sens. Environ.* 17 1–11. 10.1016/0034-4257(85)90108-7

[B3] Atzberger C. Guérif M. Baret F. Werner W. (2010). Comparative analysis of three chemometric techniques for the spectroradiometric assessment of canopy chlorophyll content in winter wheat. *Comput. Electron. Agricult.* 73 165–173. 10.1016/j.compag.2010.05.006

[B4] Bakdash J. Z. Marusich L. R. (2018). *rmcorr**: Repeated Measures Correlation. R Package Version 0.3.0.* Available online at: https://CRAN.R-project.org/package=rmcorr (accessed April 8, 2020).

[B5] Behmann J. Mahlein A.-K. Paulus S. Dupuis J. Kuhlmann H. Oerke E.-C. (2016). Generation and application of hyperspectral 3D plant models: methods and challenges. *Mach. Vis. Appl.* 27 611–624. 10.1007/s00138-015-0716-8

[B6] Behmann J. Mahlein A.-K. Paulus S. Kuhlmann H. Oerke E.-C. Plümer L. (2015). Calibration of hyperspectral close-range pushbroom cameras for plant phenotyping. *ISPRS J. Photogram. Remote Sens.* 106 172–182. 10.1016/j.isprsjprs.2015.05.010

[B7] Behmann J. Steinrücken J. Plümer L. (2014). Detection of early plant stress responses in hyperspectral images. *ISPRS J. Photogram. Remote Sens.* 93 98–111. 10.1016/j.isprsjprs.2014.03.016

[B8] Busemeyer L. Mentrup D. Möller K. Wunder E. Alheit K. Hahn V. (2013). BreedVision — A multi-sensor platform for non-destructive field-based phenotyping in plant breeding. *Sensors* 13 2830–2847. 10.3390/s130302830 23447014 PMC3658717

[B9] Ceccato P. Flasse S. Tarantola S. Jacquemoud S. Grégoire J.-M. (2001). Detecting vegetation leaf water content using reflectance in the optical domain. *Remote Sens. Environ.* 77 22–33. 10.1016/s0034-4257(01)00191-2

[B10] Charrad M. Ghazzali N. Boiteau V. Niknafs A. (2014). NbClust: an r package for determining the relevant number of clusters in a data set. *J. Statist. Soft.* 61 1–36.

[B11] Chartzoulakis K. Patakas A. Kofidis G. Bosabalidis A. Nastou A. (2002). Water stress affects leaf anatomy, gas exchange, water relations and growth of two avocado cultivars. *Scien. Horticult.* 95 39–50. 10.1016/s0304-4238(02)00016-x

[B12] Chen S. Hong X. Harris C. J. Sharkey P. M. (2004). Sparse modeling using orthogonal forward regression with PRESS statistic and regularization. *IEEE Transact. Syst. Man Cybern. Part B (Cybernetics)* 34 898–911. 10.1109/tsmcb.2003.817107 15376838

[B13] Chong I.-G. Jun C.-H. (2005). Performance of some variable selection methods when multicollinearity is present. *Chemom. Intel. Lab. Syst.* 78 103–112. 10.1016/j.chemolab.2004.12.011

[B14] Clark M. L. Roberts D. A. Clark D. B. (2005). Hyperspectral discrimination of tropical rain forest tree species at leaf to crown scales. *Remote Sens. Environ.* 96 375–398. 10.1016/j.rse.2005.03.009

[B15] Clauw P. Coppens F. Korte A. Herman D. Slabbinck B. Dhondt S. (2016). Leaf Growth response to mild drought: natural variation in arabidopsis sheds light on trait architecture. *Plant Cell* 28 2417–2434. 10.1105/tpc.16.00483 27729396 PMC5134983

[B16] Corti M. Gallina P. M. Cavalli D. Cabassi G. (2017). Hyperspectral imaging of spinach canopy under combined water and nitrogen stress to estimate biomass, water, and nitrogen content. *Biosyst. Eng.* 158 38–50. 10.1016/j.biosystemseng.2017.03.006

[B17] Daughtry C. S. T. Walthall C. L. Kim M. S. Brown De Colstoun E. Mcmurtrey Iii J. E. (2000). Estimating corn leaf chlorophyll concentration from leaf and canopy reflectance. *Remote Sens. Environ.* 74, 229–239. 10.1016/S0034-4257(00)00113-9

[B18] El-Hendawy S. E. Al-Suhaibani N. A. Elsayed S. Hassan W. M. Dewir Y. H. Refay Y. (2019). Potential of the existing and novel spectral reflectance indices for estimating the leaf water status and grain yield of spring wheat exposed to different irrigation rates. *Agricult. Water Manag.* 217 356–373. 10.1016/j.agwat.2019.03.006

[B19] Feng X. Yu C. Chen Y. Peng J. Ye L. Shen T. (2018). Non-destructive determination of shikimic acid concentration in transgenic maize exhibiting glyphosate tolerance using chlorophyll fluorescence and hyperspectral imaging. *Front. Plant Sci.* 9:468. 10.3389/fpls.2018.00468 29686693 PMC5900420

[B20] Fearn T. (2009). The effect of spectral pre-treatments on interpretation. *NIR News* 20, 15–16. 10.1255/nirn.1146

[B21] Fu P. Meacham-Hensold K. Guan K. Y. Bernacchi C. J. (2019). Hyperspectral leaf reflectance as proxy for photosynthetic capacities: an ensemble approach based on multiple machine learning algorithms. *Front. Plant Sci.* 10:730. 10.3389/fpls.2019.00730 31214235 PMC6556518

[B22] Gamon J. A. Peñuelas J. Field C. B. (1992). A narrow-waveband spectral index that tracks diurnal changes in photosynthetic efficiency. *Remote Sens. Environ.* 41 35–44. 10.1016/0034-4257(92)90059-s

[B23] Gamon J. A. Surfus J. S. (1999). Assessing leaf pigment content and activity with a reflectometer. *New Phytologist* 143, 105–117. 10.1046/j.1469-8137.1999.00424.x

[B24] Garbulsky M. F. Peñuelas J. Gamon J. Inoue Y. Filella I. (2011). The photochemical reflectance index (PRI) and the remote sensing of leaf, canopy and ecosystem radiation use efficiencies: a review and meta-analysis. *Remote Sens. Environ.* 115 281–297. 10.1016/j.rse.2010.08.023

[B25] Ge Y. Bai G. Stoerger V. Schnable J. C. (2016). Temporal dynamics of maize plant growth, water use, and leaf water content using automated high throughput RGB and hyperspectral imaging. *Comput. Electron. Agricult.* 127 625–632. 10.1016/j.compag.2016.07.028

[B26] Ge Y. F. Atefi A. Zhang H. C. Miao C. Y. Ramamurthy R. K. Sigmon B. (2019). High-throughput analysis of leaf physiological and chemical traits with VIS-NIR-SWIR spectroscopy: a case study with a maize diversity panel. *Plant Methods* 15:66. 10.1186/s13007-019-0450-8 PMC6595573 31391863

[B27] Geladi P. Kowalski B. R. (1986). Partial least-squares regression – a tutorial. *Anal. Chim. Acta* 185 1–17. 10.1016/0003-2670(86)80028-9

[B28] Gitelson A. A. Merzlyak M. N. (1997). Remote estimation of chlorophyll content in higher plant leaves. *Int. J. Remote Sens.* 18 2691–2697. 10.1080/014311697217558

[B29] Gitelson A. A. Zur Y. Chivkunova O. B. Merzlyak M. N. (2002). Assessing carotenoid content in plant leaves with reflectance spectroscopy. *Photochem. Photobiol.* 75 272–281. 10.1562/0031-8655(2002)0750272accipl2.0.co2 11950093

[B30] Granier C. Aguirrezabal L. Chenu K. Cookson S. J. Dauzat M. Hamard P. (2006). PHENOPSIS, an automated platform for reproducible phenotyping of plant responses to soil water deficit in *Arabidopsis thaliana* permitted the identification of an accession with low sensitivity to soil water deficit. *New Phytol.* 169 623–635. 10.1111/j.1469-8137.2005.01609.x 16411964

[B31] Hansen P. M. Schjoerring J. K. (2003). Reflectance measurement of canopy biomass and nitrogen status in wheat crops using normalized difference vegetation indices and partial least squares regression. *Remote Sens. Environ.* 86 542–553. 10.1016/s0034-4257(03)00131-7

[B32] Heckmann D. Schluter U. Weber A. P. M. (2017). Machine Learning techniques for predicting crop photosynthetic capacity from leaf reflectance spectra. *Mol. Plant* 10 878–890. 10.1016/j.molp.2017.04.009 28461269

[B33] Huang W. Lamb D. W. Niu Z. Zhang Y. Liu L. Wang J. (2007). Identification of yellow rust in wheat using in-situ spectral reflectance measurements and airborne hyperspectral imaging. *Precis. Agricult.* 8 187–197. 10.1007/s11119-007-9038-9

[B34] Humplík J. F. Lazár D. Fürst T. Husièková A. Spíchal L. (2015). Automated phenotyping of plant shoots using imaging methods for analysis of plant stress responses – a review. *Plant Methods* 11:29. 10.1186/s13007-015-0072-8 PMC4406171 25904970

[B35] Hunt E. R. Jr. Rock B. N. (1989). Detection of changes in leaf water content using near-infrared and middle-infrared reflectances. *Remote Sens. Environ.* 30, 43–54. 10.1016/0034-4257(89)90046-1

[B36] Inoue Y. Peñuelas J. Miyata A. Mano M. (2008). Normalized difference spectral indices for estimating photosynthetic efficiency and capacity at a canopy scale derived from hyperspectral and CO_2_ flux measurements in rice. *Remote Sens. Environ.* 112 156–172. 10.1016/j.rse.2007.04.011

[B37] Jerbi T. Wuyts N. Cane M. A. Faux P.-F. Draye X. (2015). High resolution imaging of maize (*Zea mays*) leaf temperature in the field: the key role of the regions of interest. *Funct. Plant Biol.* 42 858–864. 10.1071/fp15024 32480728

[B38] Jinwen L. Jingping Y. Pinpin F. Junlan S. Dongsheng L. Changshui G. (2009). Responses of rice leaf thickness, SPAD readings and chlorophyll a/b ratios to different nitrogen supply rates in paddy field. *Field Crops Res.* 114 426–432. 10.1016/j.fcr.2009.09.009

[B39] Kim D. M. Zhang H. Zhou H. Du T. Wu Q. Mockler T. C. (2015). Highly sensitive image-derived indices of water-stressed plants using hyperspectral imaging in SWIR and histogram analysis. *Sci. Rep.* 5:15919. 10.1038/srep15919 PMC4632122 26531782

[B40] Kim Y. Glenn D. M. Park J. Ngugi H. K. Lehman B. L. (2011). Hyperspectral image analysis for water stress detection of apple trees. *Comput. Electron. Agricult.* 77 155–160. 10.1016/j.compag.2011.04.008

[B41] Kong W. Liu F. Zhang C. Bao Y. Yu J. He Y. (2014). Fast detection of peroxidase (POD) activity in tomato leaves which infected with *Botrytis cinerea* using hyperspectral imaging. *Spectrochim. Acta Part A Mol. Biomol. Spectros.* 118 498–502. 10.1016/j.saa.2013.09.009 24080581

[B42] Korn M. Gärtner T. Erban A. Kopka J. Selbig J. Hincha D. K. (2010). Predicting *Arabidopsis* freezing tolerance and heterosis in freezing tolerance from metabolite composition. *Mol. Plant* 3 224–235. 10.1093/mp/ssp105 20026477 PMC2807929

[B43] Krishna G. Sahoo R. N. Singh P. Bajpai V. Patra H. Kumar S. (2019). Comparison of various modelling approaches for water deficit stress monitoring in rice crop through hyperspectral remote sensing. *Agricult. Water Manag.* 213 231–244. 10.1016/j.agwat.2018.08.029

[B44] Kuhn M. Wing J. Weston S. Williams A. Keefer C. Engelhardt A. (2018). *Caret: Classification and Regression Training. R Package Version 6.0-79.* Available online at: https://CRAN.R-project.org/package=caret (accessed April 8, 2020).

[B45] Leinonen I. Jones H. G. (2004). Combining thermal and visible imagery for estimating canopy temperature and identifying plant stress. *J. Exp. Bot.* 55 1423–1431. 10.1093/jxb/erh146 15133055

[B46] Liang J. Zia A. Zhou J. Sirault X. (2013). “3D plant modelling via hyperspectral imaging,” in *Proceedings of the 2013 IEEE International Conference on Computer Vision Workshops (ICCVW)*, (Piscataway, NJ: IEEE), 172–177.

[B47] Liaw A. Wiener M. (2002). Classification and regression by randomForest. *R News* 2 18–22.

[B48] Liu K. Goodman M. Muse S. Smith J. S. Buckler E. Doebley J. (2003). Genetic structure and diversity among maize inbred lines as inferred from DNA microsatellites. *Genetics* 165 2117–2128. 14704191 10.1093/genetics/165.4.2117 PMC1462894

[B49] Maai E. Shimada S. Yamada M. Sugiyama T. Miyake H. Taniguchi M. (2011). The avoidance and aggregative movements of mesophyll chloroplasts in C4 monocots in response to blue light and abscisic acid. *J. Exp. Bot.* 62 3213–3221. 10.1093/jxb/err008 21339388

[B50] Mcburney T. (1992). The relationship between leaf thickness and plant water potential. *J. Exp. Bot.* 43 327–335. 10.1093/jxb/43.3.327

[B51] Merilo E. Yarmolinsky D. Jalakas P. Parik H. Tulva I. Rasulov B. (2018). Stomatal VPD response: there is more to the story than ABA. *Plant Physiol.* 176 851–864. 10.1104/pp.17.00912 28986421 PMC5761775

[B52] Mevik B.-H. Wehrens R. Liland K. H. (2016). *pls: Partial Least Squares and Principal Component Regression. R Package Version 2.6-0.* Available online at: https://CRAN.R-project.org/package=pls (accessed April 7, 2020).

[B53] Mishra P. Lohumi S. Khan H. A. Nordon A. (2020a). Close-range hyperspectral imaging of whole plants for digital phenotyping: recent applications and illumination correction approaches. *Comput. Electron. Agricult.* 178:105780.

[B54] Mishra P. Polder G. Gowen A. Rutledge D. N. Roger J. M. (2020b). Utilising variable sorting for normalisation to correct illumination effects in close-range spectral images of potato plants. *Biosyst. Eng.* 197 318–323. 10.1016/j.biosystemseng.2020.07.010

[B55] Moghimi A. Yang C. Miller M. E. Kianian S. F. Marchetto P. M. (2018). A novel approach to assess salt stress tolerance in wheat using hyperspectral imaging. *Front. Plant Sci.* 9:1182. 10.3389/fpls.2018.01182 30197650 PMC6117507

[B56] Neilson E. H. Edwards A. M. Blomstedt C. K. Berger B. Møller B. L. Gleadow R. M. (2015). Utilization of a high-throughput shoot imaging system to examine the dynamic phenotypic responses of a C4 cereal crop plant to nitrogen and water deficiency over time. *J. Exp. Bot.* 66 1817–1832. 10.1093/jxb/eru526 25697789 PMC4378625

[B57] Odilbekov F. Armoniene R. Henriksson T. Chawade A. (2018). Proximal Phenotyping and machine learning methods to identify septoria tritici blotch disease symptoms in wheat. *Front. Plant Sci.* 9:685. 10.3389/fpls.2018.00685 29875788 PMC5974968

[B58] Osco L. P. Ramos A. P. M. Pinheiro M. M. F. Moriya E. A. S. Imai N. N. Estrabis N. (2020). A machine learning framework to predict nutrient content in valencia-orange leaf hyperspectral measurements. *Remote Sens.* 12:906. 10.3390/rs12060906

[B59] Pandey P. Ge Y. Stoerger V. Schnable J. C. (2017). High throughput *in vivo* analysis of plant leaf chemical properties using hyperspectral imaging. *Front. Plant Sci.* 8:1348. 10.3389/fpls.2017.01348 PMC5540889 28824683

[B60] Peñuelas J. Filella I. Biel C. Serrano L. Savé R. (1993). The reflectance at the 950-970 nm region as an indicator of plant water status. *Int. J. Remote Sens.* 14 1887–1905. 10.1080/01431169308954010

[B61] Peñuelas J. Filella I. Gamon J. A. (1995). Assessment of photosynthetic radiation-use efficiency with spectral reflectance. *New Phytologist* 131, 291–296. 10.1111/j.1469-8137.1995.tb03064.x

[B62] R Core Team (2015). *R: A Language and Environment for Statistical Computing.* Vienna: The R Foundation for Statistical Computing.

[B63] R Core Team (2017). *R:A Language and Environment for Statistical Computing.* Vienna: The R Foundation for Statistical Computing.

[B64] Rapaport T. Hochberg U. Shoshany M. Karnieli A. Rachmilevitch S. (2015). Combining leaf physiology, hyperspectral imaging and partial least squares-regression (PLS-R) for grapevine water status assessment. *Isp. J. Photogram. Remote Sens.* 109 88–97. 10.1016/j.isprsjprs.2015.09.003

[B65] Ritchie S. W. Hanway J. J. Thompson H. E. (1996). *How A Corn Plant Develops*. Special Report No. 48. Ames, IA: Iowa State University of Science and Technology, Cooperative Extension Service.

[B66] Römer C. Wahabzada M. Ballvora A. Pinto F. Rossini M. Panigada C. (2012). Early drought stress detection in cereals: simplex volume maximisation for hyperspectral image analysis. *Funct. Plant Biol.* 39 878–890. 10.1071/fp12060 32480838

[B67] Roscher R. Behmann J. Mahlein A.-K. Dupuis J. Kuhlmann H. Plümer L. (2016). Detection of disease symptoms on hyperspectral 3D plant models. *ISPRS Ann. Photogram. Remote Sens. Spat. Inform. Sci.* 3 89–96. 10.5194/isprs-annals-iii-7-89-2016

[B68] Rouse J. W. Jr. Haas R. H. Schell J. A. Deering D. W. (1974). “Monitoring vegetation systems in the Great Plains with ERTS,” in *Third ERTS Symposium, NASA SP-351* (Washington DC), 309–317.

[B69] Sancho-Knapik D. Álvarez-Arenas T. G. Peguero-Pina J. J. Fernández V. Gil-Pelegrín E. (2011). Relationship between ultrasonic properties and structural changes in the mesophyll during leaf dehydration. *J. Exp. Bot.* 62 3637–3645. 10.1093/jxb/err065 21414961

[B70] Sandmeier S. Müller C. Hosgood B. Andreoli G. (1998). Physical mechanisms in hyperspectral BRDF data of grass and watercress. *Remote Sens. Environ.* 66 222–233. 10.1016/s0034-4257(98)00060-1

[B71] Scippa G. S. Di Michele M. Onelli E. Patrignani G. Chiatante D. Bray E. A. (2004). The histone-like protein H1-S and the response of tomato leaves to water deficit. *J. Exp. Bot.* 55 99–109. 10.1093/jxb/erh022 14645393

[B72] Serrano L. Peñuelas J. Ustin S. L. (2002). Remote sensing of nitrogen and lignin in Mediterranean vegetation from AVIRIS data: decomposing biochemical from structural signals. *Remote Sens. Environ.* 81 355–364. 10.1016/s0034-4257(02)00011-1

[B73] Simko I. Hayes R. J. Furbank R. T. (2016). Non-destructive phenotyping of lettuce plants in early stages of development with optical sensors. *Front. Plant Sci.* 7:1985. 10.3389/fpls.2016.01985 28083011 PMC5187177

[B74] Sims D. A. Gamon J. A. (2002). Relationships between leaf pigment content and spectral reflectance across a wide range of species, leaf structures and developmental stages. *Remote Sens. Environ.* 81 337–354. 10.1016/s0034-4257(02)00010-x

[B75] Slaton M. R. Hunt E. R. Jr. Smith W. K. (2001). Estimating near-infrared leaf reflectance from leaf structural characteristics. *Am. J. Bot.* 88 278–284. 10.2307/2657019 11222250

[B76] Sun C. X. Li C. C. Zhang C. Y. Hao L. Y. Song M. Liu W. (2018). Reflectance and biochemical responses of maize plants to drought and re-watering cycles. *Ann. Appl. Biol.* 172 332–345. 10.1111/aab.12423

[B77] Susič N. Žibrat U. Širca S. Strajnar P. Razinger J. Knapic M. (2018). Discrimination between abiotic and biotic drought stress in tomatoes using hyperspectral imaging. *Sens. Actuat. B Chem.* 273 842–852. 10.1016/j.snb.2018.06.121

[B78] Syvertsen J. P. Levy Y. (1982). Diurnal changes in citrus leaf thickness, leaf water potential and leaf to air temperature difference. *J. Exp. Bot.* 33 783–789. 10.1093/jxb/33.4.783 12432039

[B79] Thomas S. Behmann J. Steier A. Kraska T. Muller O. Rascher U. (2018). Quantitative assessment of disease severity and rating of barley cultivars based on hyperspectral imaging in a non-invasive, automated phenotyping platform. *Plant Methods* 14:45. 10.1186/s13007-018-0313-8 PMC5994119 29930695

[B80] Tirado S. B. St Dennis S. Enders T. A. Springer N. M. (2020). Utilizing top-down hyperspectral imaging for monitoring genotype and growth conditions in maize. *BioRxiv* [Preprint]. 10.1101/2020.01.21.914069

[B81] Vigneau N. Ecarnot M. Rabatel G. Roumet P. (2011). Potential of field hyperspectral imaging as a non destructive method to assess leaf nitrogen content in wheat. *Field Crops Res.* 122 25–31. 10.1016/j.fcr.2011.02.003

[B82] Virlet N. Sabermanesh K. Sadeghi-Tehran P. Hawkesford M. J. (2017). Field Scanalyzer: an automated robotic field phenotyping platform for detailed crop monitoring. *Funct. Plant Biol.* 44 143–153. 10.1071/fp16163 32480553

[B83] Wold S. Sjöström M. Eriksson L. (2001). PLS-regression: a basic tool of chemometrics. *Chemometr. Intel. Lab. Syst.* 58 109–130. 10.1016/s0169-7439(01)00155-1

[B84] Wuyts N. Massonnet C. Dauzat M. Granier C. (2012). Structural assessment of the impact of environmental constraints on *Arabidopsis thaliana* leaf growth: a 3D approach. *Plant Cell Environ.* 35 1631–1646. 10.1111/j.1365-3040.2012.02514.x 22471732

[B85] Yamada M. Kawasaki M. Sugiyama T. Miyake H. Taniguchi M. (2009). Differential positioning of C4 mesophyll and bundle sheath chloroplasts: aggregative movement of C4 mesophyll chloroplasts in response to environmental stresses. *Plant Cell Physiol.* 50 1736–1749. 10.1093/pcp/pcp116 19667101

[B86] Yang W. N. Guo Z. L. Huang C. L. Duan L. F. Chen G. X. Jiang N. (2014). Combining high-throughput phenotyping and genome-wide association studies to reveal natural genetic variation in rice. *Nat. Commun.* 5:5087. 10.1038/ncomms6087 25295980 PMC4214417

[B87] Yendrek C. R. Tomaz T. Montes C. M. Cao Y. Morse A. M. Brown P. J. (2017). High-throughput phenotyping of maize leaf physiological and biochemical traits using hyperspectral reflectance. *Plant Physiol.* 173 614–626. 10.1104/pp.16.01447 28049858 PMC5210743

[B88] Yi Q.-X. Bao A.-M. Wang Q. Zhao J. (2013). Estimation of leaf water content in cotton by means of hyperspectral indices. *Comput. Electron. Agricult.* 90 144–151. 10.1016/j.compag.2012.09.011

[B89] Yu G.-R. Miwa T. Nakayama K. Matsuoka N. Kon H. (2000). A proposal for universal formulas for estimating leaf water status of herbaceous and woody plants based on spectral reflectance properties. *Plant Soil* 227 47–58.

[B90] Zhang C. Filella I. Garbulsky M. F. Peñuelas J. (2016). Affecting factors and recent improvements of the photochemical reflectance index (PRI) for remotely sensing foliar, canopy and ecosystemic radiation-use efficiencies. *Remote Sens.* 8:677. 10.3390/rs8090677

[B91] Zhang X. He Y. (2013). Rapid estimation of seed yield using hyperspectral images of oilseed rape leaves. *Industr. Crops Product.* 42 416–420. 10.1016/j.indcrop.2012.06.021

[B92] Žibrat U. Susič N. Knapič M. Širca S. Strajnar P. Razinger J. (2019). Pipeline for imaging, extraction, pre-processing, and processing of time-series hyperspectral data for discriminating drought stress origin in tomatoes. *MethodsX* 6 399–408. 10.1016/j.mex.2019.02.022 30886829 PMC6402290

[B93] Zhu Y. Yao X. Tian Y. Liu X. Cao W. (2008). Analysis of common canopy vegetation indices for indicating leaf nitrogen accumulations in wheat and rice. *Int. J. Appl. Earth Obs. Geoinf.* 10, 1–10. 10.1016/j.jag.2007.02.006

