# Corrigendum: Proximal hyperspectral imaging detects diurnal and drought-induced changes in maize physiology

**DOI:** 10.3389/fpls.2024.1379654

**Published:** 2024-02-21

**Authors:** Stien Mertens, Lennart Verbraeken, Heike Sprenger, Kirin Demuynck, Katrien Maleux, Bernard Cannoot, Jolien De Block, Steven Maere, Hilde Nelissen, Gustavo Bonaventure, Steven J. Crafts-Brandner, Jonathan T. Vogel, Wesley Bruce, Dirk Inzé, Nathalie Wuyts

**Affiliations:** ^1^ Department of Plant Biotechnology and Bioinformatics, Ghent University, Ghent, Belgium; ^2^ VIB-UGent Center for Plant Systems Biology, Ghent, Belgium; ^3^ BASF SE, Ghent, Belgium; ^4^ BASF Corporation, Research Triangle Park, NC, United States

**Keywords:** automated phenotyping platform, hyperspectral, phenotyping, drought, physiology, maize, proximal sensing

In the published article, there was an error in **Figure 2** and **Supplementary Figure 1**. The values on the left Y-axis in **Figure 2** and **Supplementary Figure 1** were switched. The corrected **Figure 2** and its caption appear below.

**Figure 2:**
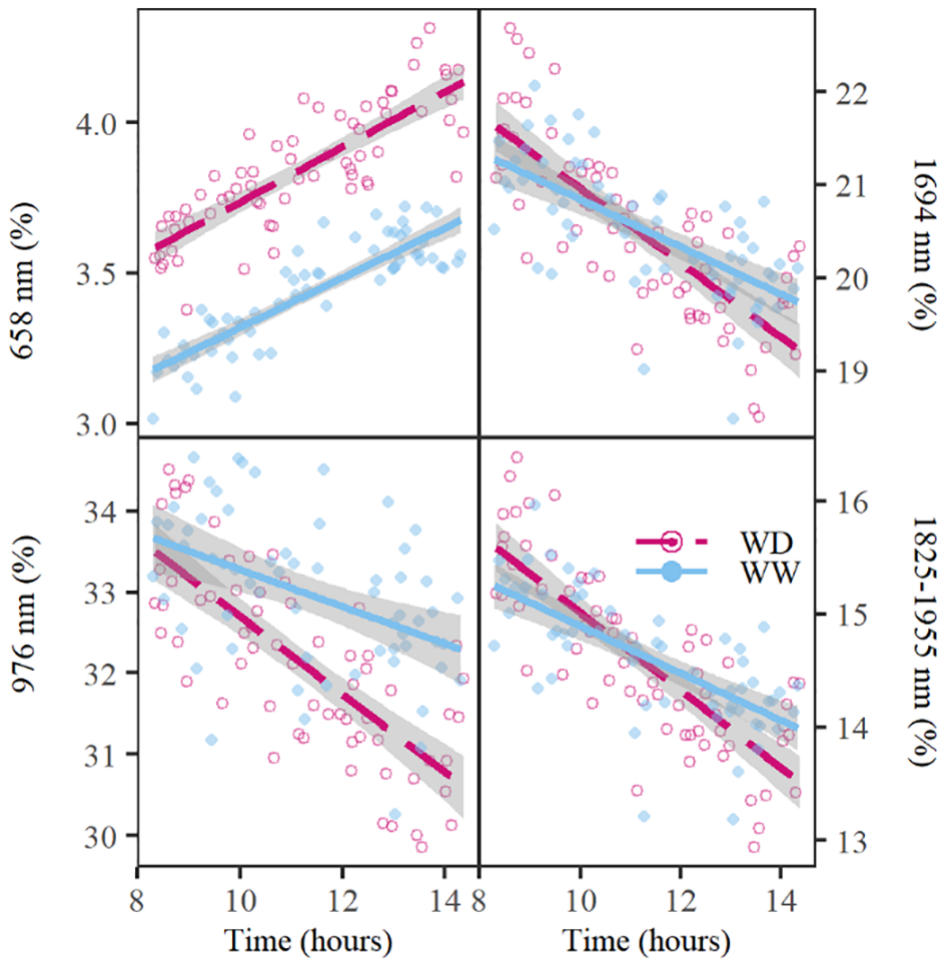
Diurnal changes in relative reflectance at 658; 976; and 1,694 nm and the water absorption trough with the ridge at 1,825 nm and the valley at 1,955 nm on day 6 of the drought period. The well-watered (WW) and water deficit (WD) treatments are indicated with a blue line or dot and a red dashed line or circle, respectively. The lines show the average trend of the treatment, whereas the dots and circles represent the relative reflectance of individual plants at the respective wavelengths. The gray shading around the lines indicate the standard error of relative reflectance. The water absorption trough depth values were calculated as the difference in relative reflectance between 1,825 and 1,955 nm.

The authors apologize for this error and state that this does not change the scientific conclusions of the article in any way. The original article has been updated.

