# Supplementary material for: Proximal Hyperspectral Imaging Detects Diurnal and Drought-Induced Changes in Maize Physiology
Source: Front Plant Sci. 2021 Feb 22;12:640914. doi: 10.3389/fpls.2021.640914 (PMC7937976; doi:10.3389/fpls.2021.640914)
Supplement: Supplementary Figure 1 — Time-of-day effects on relative reflectance at 551, 708, 721, 1482, 1937, 2110 and 2321 nm, and the water absorption trough depths. [file Data_Sheet_1.pdf]

**Supplemental Figures and Legends**

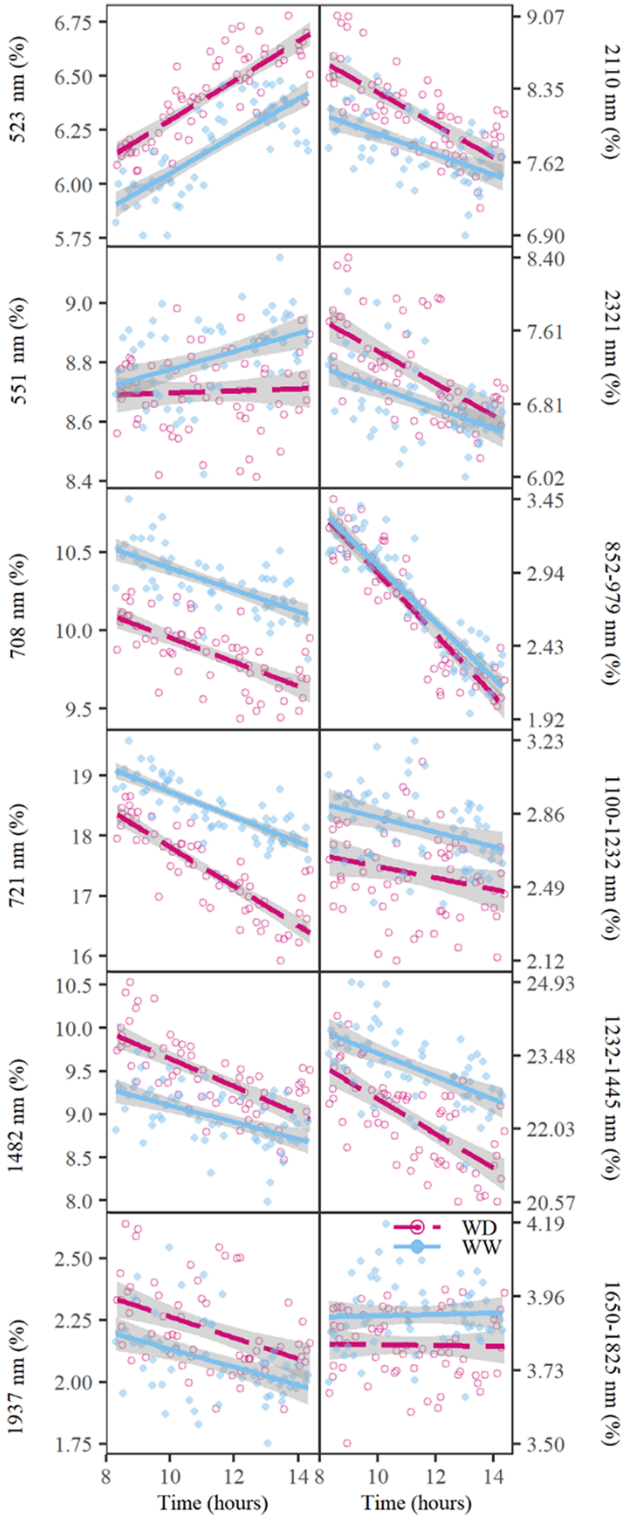


**Supplemental Figure S1.** Time-of-day effects on relative reflectance at 551, 708, 721, 1482, 1937, 2110 and 2321 nm, and the water absorption trough depths with highest wavelengths of the trough at 852, 1100, 1232, 1650 and 1825 nm and the lowest at 979, 1232, 1445, 1825 and 1955 nm. The well-watered (WW) and water-deficit (WD) treatments are indicated with a blue line or dot, and a red-pink dashed line or circle, respectively. The water absorption trough depths were calculated as the difference in relative reflectance between the first and second wavelength.

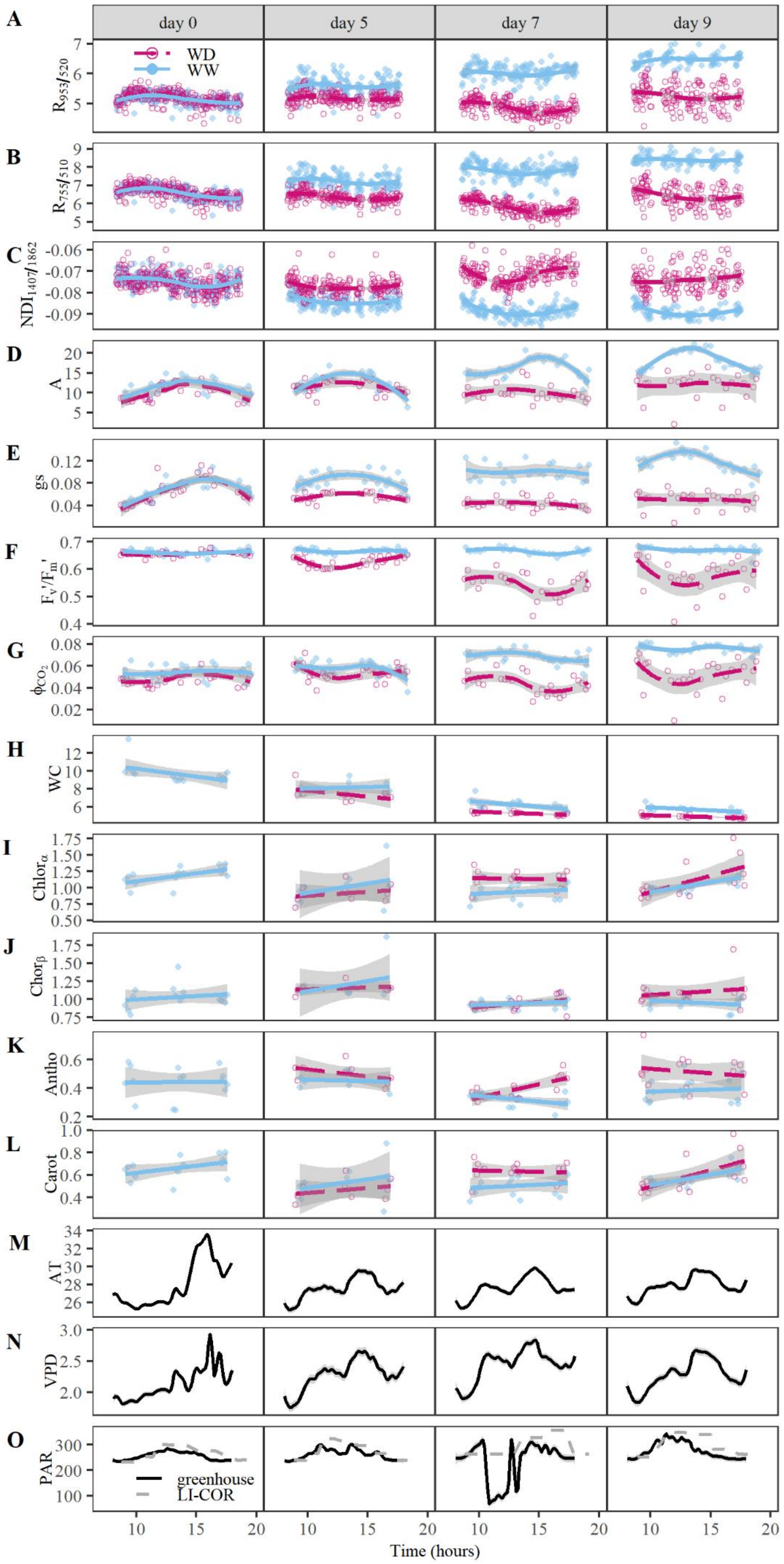

A
B
C
D
E
F
G
H
I
J
K
L
M
N
O
day 0
day 5
day 7
day 9
WD
WW
$R_{953}/_{520}$
$R_{755}/_{510}$
$NDI_{1407}/_{1862}$
A
gs
$F_v'/F_m'$
$\phi_{CO_2}$
WC
$Chlor_\alpha$
$Chor_\beta$
Antho
Carot
AT
VPD
PAR
greenhouse
LI-COR
Time (hours)

**Supplemental Figure S2.** The responses of physiological traits and indices to diurnal variations in environmental conditions and drought. Physiological trait measurements and indices of well-watered (WW) and water-deficit (WD) plants were compared during the drought period (day 0, 5, 7 and 9). The average trends of the WW and WD treatments are indicated by a blue line and red-pink dashed line, respectively. The measurements on individual plants of the treatments are visualized with a blue dot (WW) or red circle (WD). **A**, Index $R_{953/520}$. **B**, Index $R_{755/510}$. **C**, Index $NDI_{1407/1860}$. **D**, Photosynthetic rate (A, μmol $CO_2$ $m^{-2}$ $s^{-1}$). **E**, Stomatal conductance ($g_s$, mol $H_2O$ $m^{-2}$ $s^{-1}$). **F**, Efficiency of energy harvesting by oxidized PS2 reaction centers in the light ($F_v$'/$F_m$'). **G**, Quantum yield based on $CO_2$ ($\Phi_{CO2}$). **H**, Leaf water content (WC, g $H_2O$/g dry weight). **I**, Chlorophyll α content ($Chlor_\alpha$, mg/g fresh weight). **J**, Chlorophyll β content ($Chlor_\beta$, mg/g fresh weight). **K**, Anthocyanin content (Antho, anthocyanin/mg fresh weight). **L**, Carotenoid content (Carot, mg/g fresh weight). **M**, Average greenhouse air temperature (AT, °C). **N**, Vapor pressure deficit (VPD, kPa). **O**, Photosynthetically active radiation (PAR, μmol photons $m^{-2}$ $s^{-1}$). In **M**, **N** and **O**, the black line represents the greenhouse environmental conditions, which is supplemented in **O** with the PAR settings of the LI-COR LI-6400 (gray dashed line). The gray shading around the lines indicates the standard error of relative reflectance, physiological traits and environmental conditions.

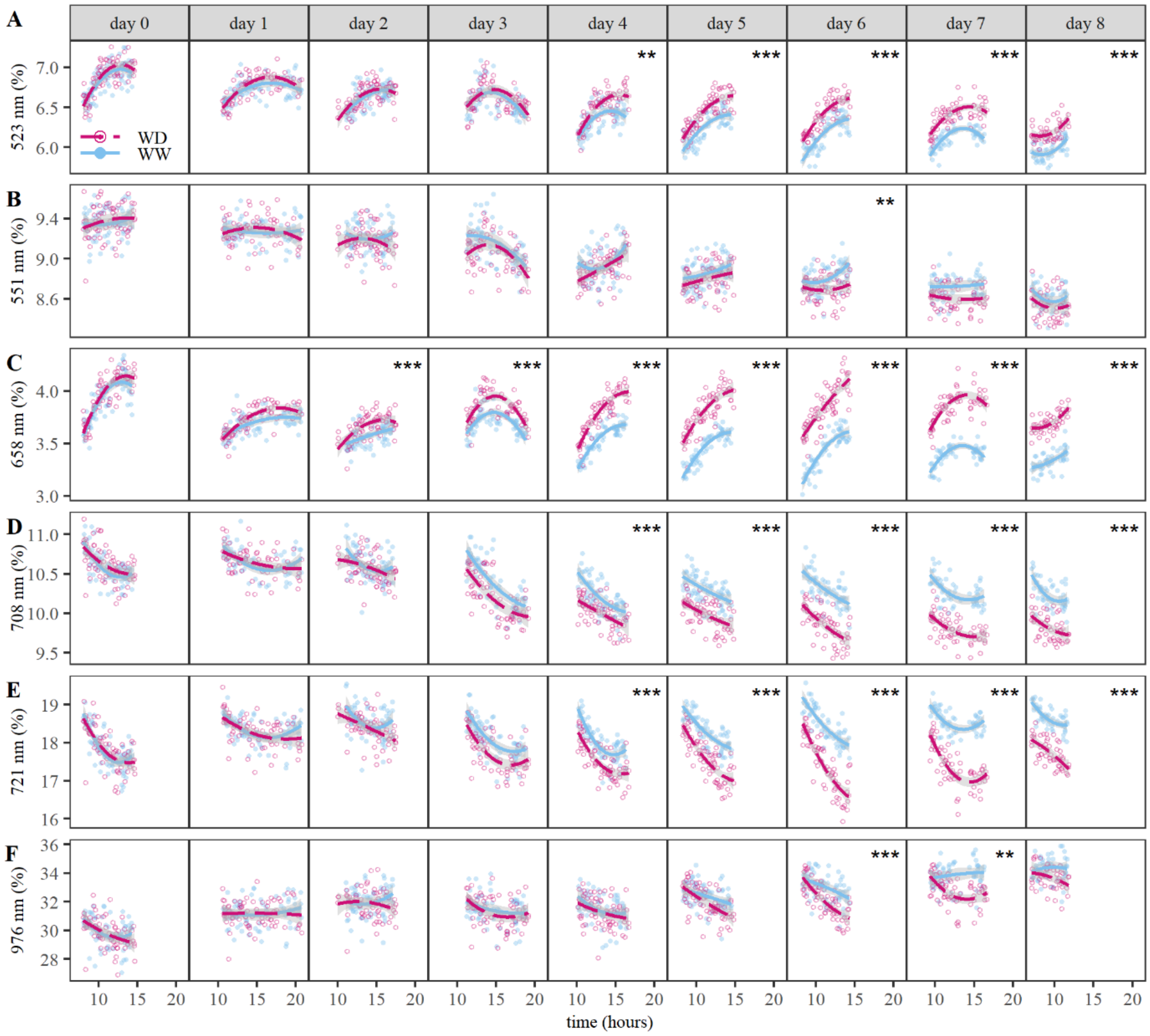


**Supplemental Figure S3**. Drought differences in VNIR reflectance. Six VNIR wavelengths that are not strongly correlated with each other (r<0.8) were selected for this analysis. Relative reflectance of well-watered (WW, blue dots) and water-deficit plants (WD, red-pink circles) were plotted for the whole drought period from day 0 to 8. Acute drought started at the end of day 0 until day 7. WD plants were watered again to maintain drought soil water content from day 8 onwards. The blue line and red-pink dashed line indicate the average diurnal trends of the WW and WD treatment, respectively. The wavelengths visualized in this graph are: **A**, blue-green at 523 nm, **B**, green at 551 nm, **C**, red at 658 nm, **D**, red-edge at 708 nm, **E**, red-edge at 721 nm and **F**, NIR at 976 nm. Significant differences between WW and WD are indicated (two-tailed Student's t-test, **: P<0.01, ***: P<0.001).

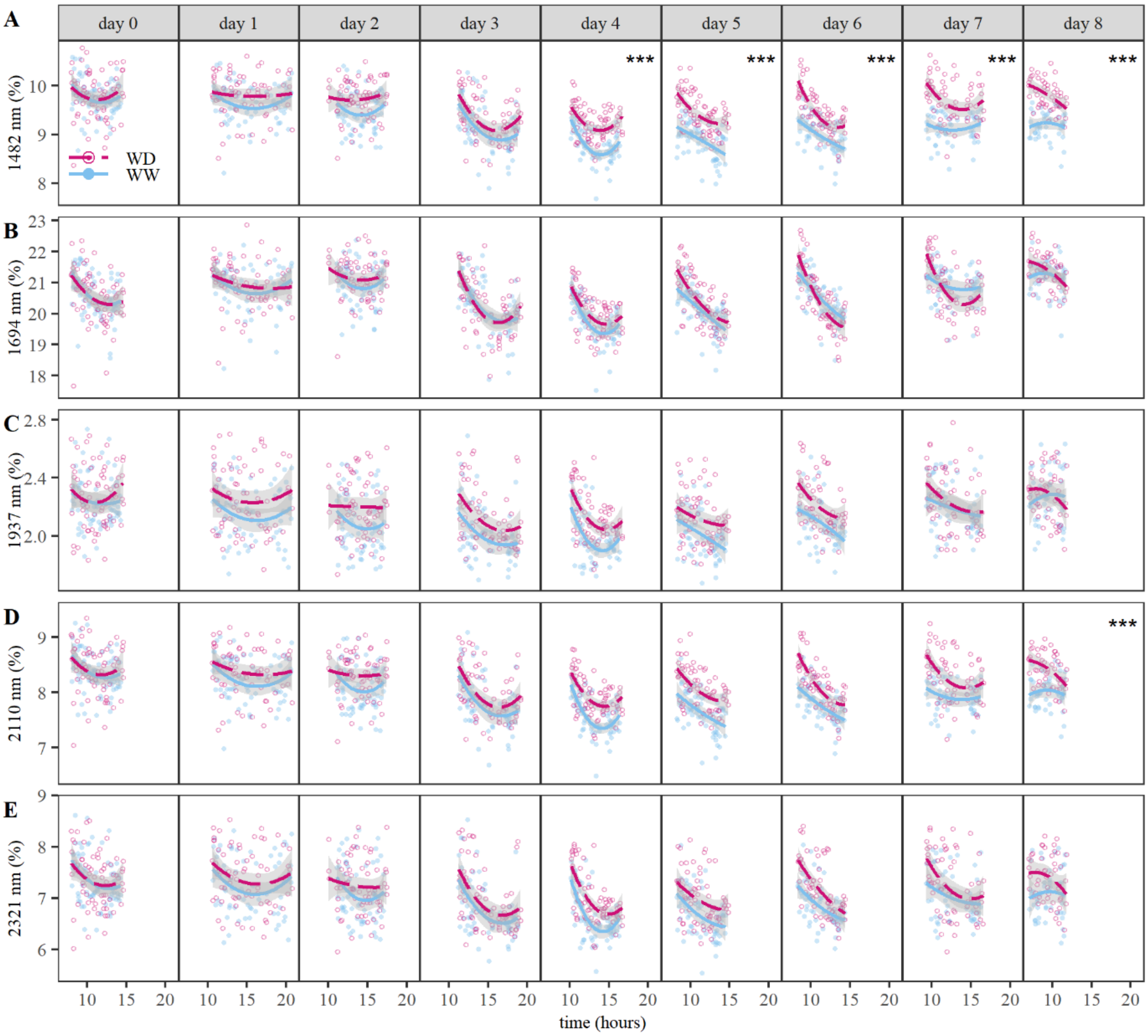


**Supplemental Figure S4.** Drought differences in SWIR reflectance. Five SWIR wavelengths that are not strongly correlated with each other (r<0.8) were selected for this analysis. Relative reflectance of well-watered (WW, blue dots) and water-deficit plants (WD, red-pink circles) were plotted for the whole drought period from day 0 to 8. Acute drought started at the end of day 0 until day 7. WD plants were watered again to maintain drought soil water content from day 8 onwards. The blue line and red-pink dashed line indicate the average diurnal trends of the WW and WD treatment, respectively. The wavelengths visualized in this graph are: **A**, water absorption trough with the lowest value at 1482 nm, **B**, 1694 nm, **C**, water absorption trough with the lowest value at 1937 nm, **D**, 2110 nm, and **E**, 2321 nm. Significant differences between WW and WD are indicated (two-tailed Student's t-test, ***: P<0.001).

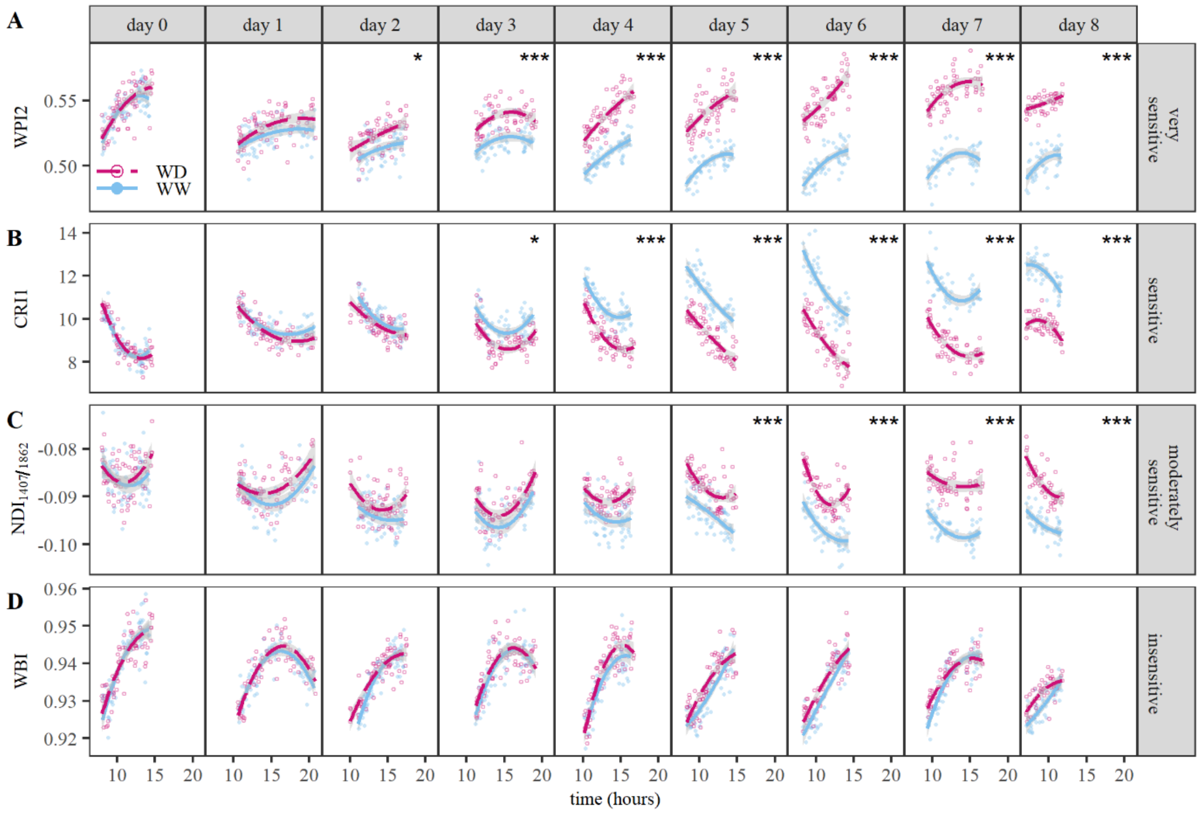


**Supplemental Figure S5.** The detection of drought effects using standard indices. The indices were subdivided into four groups based on their drought sensitivity: very sensitive, sensitive, moderately sensitive and insensitive. One index of each group is visualized in this figure. **A-D**, Shown are the ability to detect drought of the very sensitive WPI2 index (**A**), the sensitive CRI1 index (**B**), the moderately sensitive $NDI_{1407/1862}$ index (**C**) and the insensitive WBI index (**D**). The average diurnal trends of the well-watered (WW) and water-deficit (WD) treatments are represented by a blue line and red-pink dashed line, respectively. The gray shading around the line indicates the standard error. To illustrate the variation within the treatments, individual plants were plotted with blue dots (WW) and red-pink circles (WD). Acute drought started at the end of day 0 until day 7. WD plants received water up to the drought soil water content from day 8 onwards. Significant differences between WW and WD are indicated (two-tailed Student's t-test, *: $P<0.05$, ***: $P<0.001$).

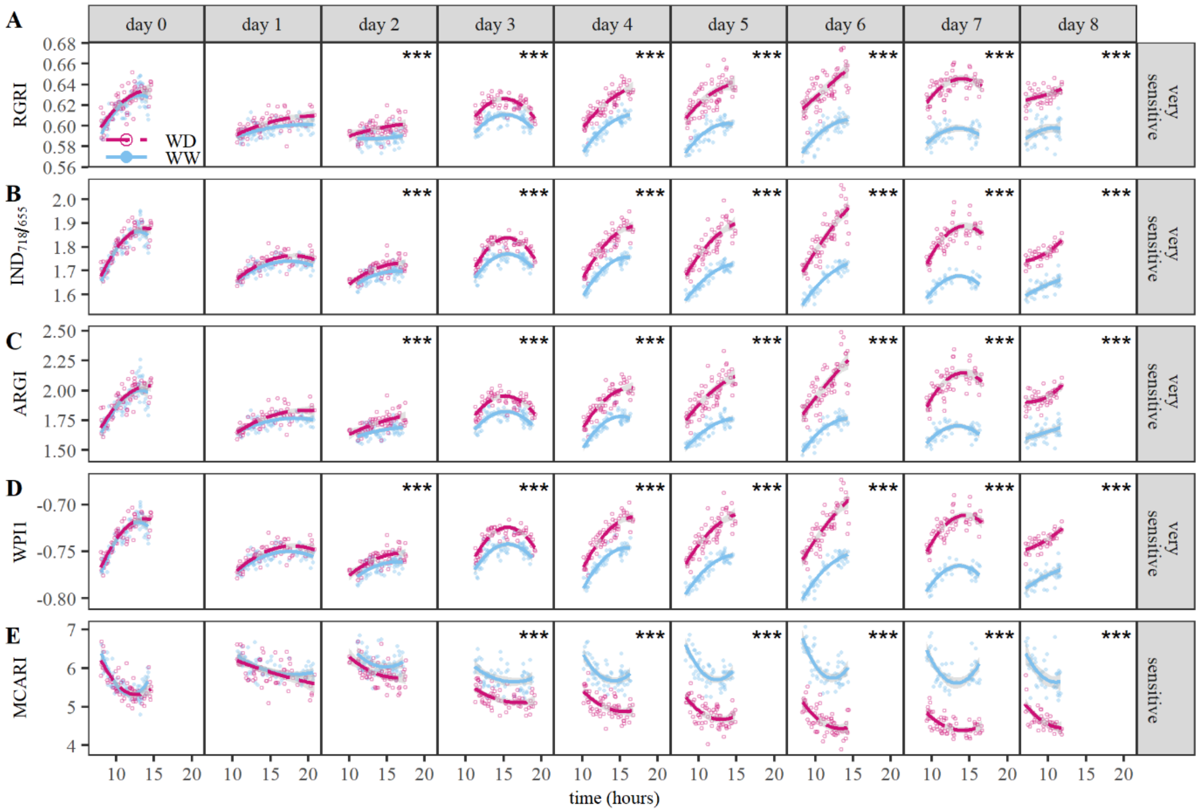


**Supplemental Figure S6.** The detection of drought effects using very sensitive and sensitive indices. The indices were subdivided into four groups based on their drought sensitivity: very sensitive, sensitive, moderately sensitive and insensitive. The sensitivity group of these indices is indicated in the gray bars at the right side of the rows. Index values for well-watered (WW, blue dots) and water-deficit plants (WD, red circles) were plotted for the whole drought period from day 0 to 8. Acute drought started at the end of day 0 until day 7. WD plants were watered again to maintain drought soil water content from day 8 onwards. The blue line and red-pink dashed line indicate the average diurnal trends of the WW and WD treatment, respectively. The indices visualized in this graph are: **A**, RGRI, **B**, $IND_{715/655}$, **C**, ARGI, **D**, WPI1, and **E**, MCARI. Significant differences between WW and WD are indicated (two-tailed Student's t-test, ***: $P<0.001$).

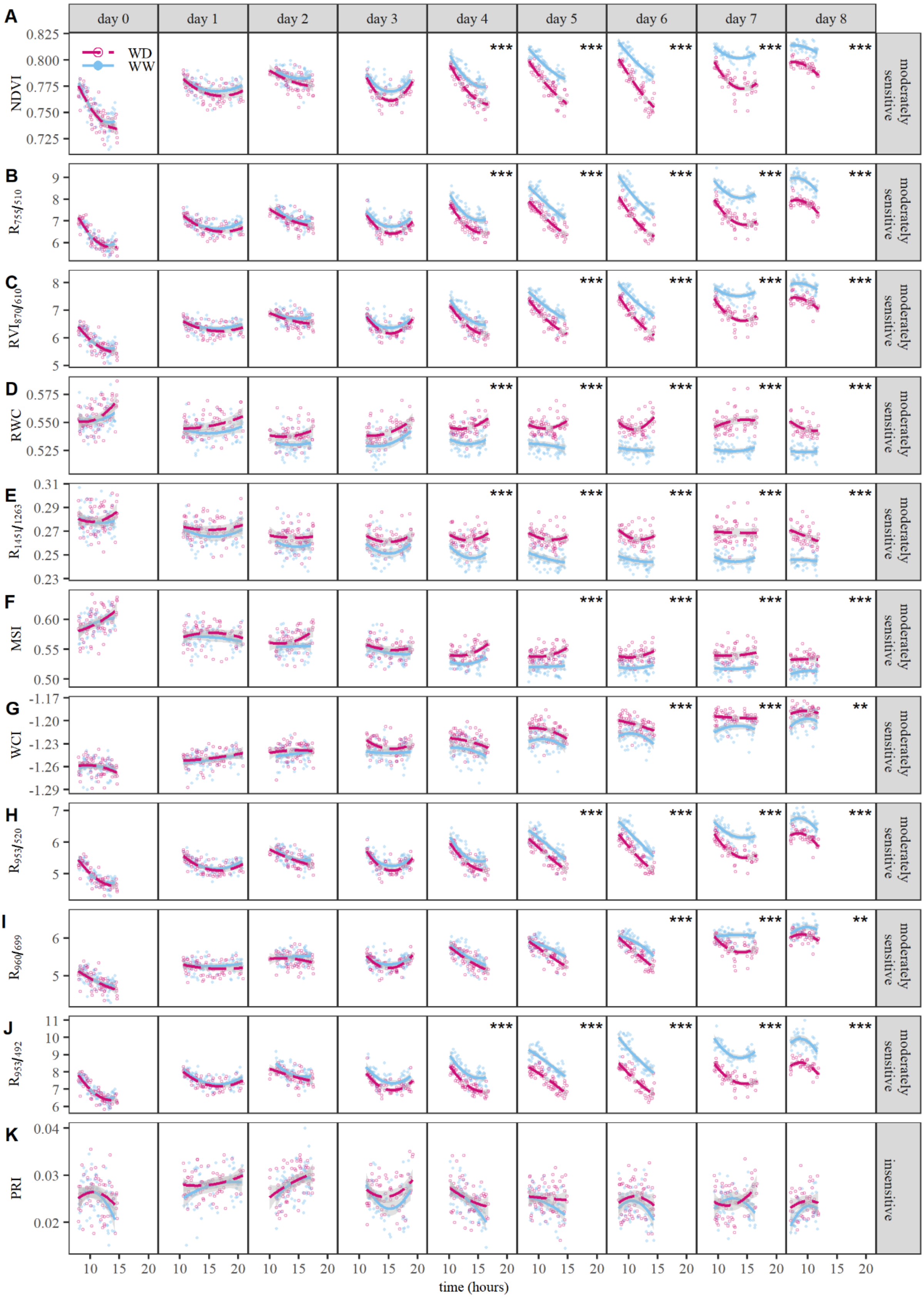


**Supplemental Figure S7.** The detection of drought effects of moderately sensitive and insensitive indices. The indices were subdivided into four groups based on their drought sensitivity: very sensitive, sensitive, moderately sensitive and insensitive. The sensitivity group of these indices is indicated in the gray bars at the right side of the rows. Index values

for well-watered (WW, blue dots) and water-deficit plants (WD, red-pink circles) were plotted for the whole drought period from day 0 to 8. Acute drought started at the end of day 0 until day 7. WD plants were watered again to maintain drought soil water content from day 8 onwards. The blue line and red-pink dashed line indicate the average diurnal trends of the WW and WD treatment, respectively. The indices visualized in this graph are: **A**, NDVI, **B**, $R_{755/510}$, **C**, $RVI_{870/610}$, **D**, RWC, **E**, $R_{1451/1263}$, **F**, MSI, **G**, WCI, **H**, $R_{953/520}$, **I**, $R_{960/699}$, **J**, $R_{953/492}$, **K**, PRI. Significant differences between WW and WD are indicated (two-tailed Student's t-test, ***: $P<0.001$).
