# Supplementary material for: Proximal Hyperspectral Imaging Detects Diurnal and Drought-Induced Changes in Maize Physiology
Source: Front Plant Sci. 2021 Feb 22;12:640914. doi: 10.3389/fpls.2021.640914 (PMC7937976; doi:10.3389/fpls.2021.640914)
Supplement: Supplementary Table 1 — The representative wavelengths and the wavelength ranges that are most strongly correlated with them. [file Data_Sheet_2.PDF]

Supplemental Tables

Supplemental Table S1. The representative wavelengths and the wavelength ranges that are most strongly correlated with them (Pearson correlation test, r>0.8).

Representative wavelengths	Correlated wavelengths
**523**	514 – 531<br>573 – 613<br>699 – 701
**551**	532 – 572<br>702 – 704
**658**	480 – 513<br>614 – 698
**708**	705 – 714
**721**	715 – 733
**976**	733 – 1005
**1482**	1407 – 1556
**1694**	1006 – 1400<br>1557 – 1867
**1937**	1906 – 2004<br>2414 – 2470
**2110**	1401 – 1406<br>1868 – 1898<br>2005 – 2252
**2321**	1899 – 1905<br>2253 – 2413

Supplemental Table S2. The relationship between relative reflectance and physiological traits. The slope for the water-deficit (WD) treatment and the interaction between relative reflectance and the drought treatment for each physiological trait are shown in this table. The physiological traits are photosynthetic rate (A, μmol CO_2 m^{-2} s^{-1}), transpiration rate (E, mmol H_2O m^{-2} s^{-1}), efficiency of energy harvesting by oxidized PS2 reaction centers in the light (F_v'/F_m'), stomatal conductance (g_s, mol H_2O m^{-2} s^{-1}), quantum yield based on CO_2 (Φ_{CO2}), effective quantum yield of photosystem 2 (Φ_{PS2}), leaf water content (WC, g H_2O/g dry weight), leaf water potential (ψ, MPa). The unit of the wavelengths is nm. Significant slopes and interactions are indicated (two-tailed Student's t-test, *: P<0.05, **: P<0.01, ***: P<0.001).

**Trait**	**Effect**	**523**	**551**	**658**	**708**	**721**	**976**	**1482**	**1694**	**1937**	**2110**	**2321**
**A**	Slope WD	-2.838	-2.905	-0.192	-1.212	0.353	0.892***	0.609	0.930*	-0.986	0.599	0.426
	Interaction	-8.387***	-8.458**	-6.234*	2.926	3.526***	0.330	0.721	1.450**	-0.691	0.863	0.084
**E**	Slope WD	0.071	-0.493	0.167	-0.339	-0.112	0.044	0.080	0.019	-0.052	0.026	-0.028
	Interaction	-1.703***	-1.206*	-0.425	0.426	0.822***	0.203***	0.147	0.466***	-0.754*	0.151	0.010
**F_v'/F_m'**	Slope WD	-0.217***	0.222***	-0.133***	0.139***	0.064***	-0.002	-0.022	0.015*	-0.118***	-0.009	-0.005
	Interaction	0.204***	-0.222***	0.098***	-0.133***	-0.059***	0.003	0.023	-0.013	0.112***	0.010	0.005
**g_s**	Slope WD	-0.041***	0.042**	-0.024**	0.027**	0.010***	-0.002	-0.000	0.002	-0.021*	0.000	-0.002
	Interaction	-0.037*	-0.121***	-0.024	-0.025	0.017***	0.010***	0.011	0.015***	0.007	0.010	0.004
**ϕ_{CO2}**	Slope WD	-0.020***	-0.002	-0.009**	0.002	0.006***	0.004***	0.001	0.005***	-0.010*	0.002	0.002
	Interaction	-0.016*	-0.036***	-0.029**	0.004	0.008***	0.000	0.003	0.002	0.006	0.003	0.001
**ϕ_{PS2}**	Slope WD	-0.270***	0.307***	-0.174***	0.189***	0.083***	-0.005	-0.029	0.017	-0.156***	-0.013	-0.009
	Interaction	0.291***	-0.283***	0.149***	-0.173***	-0.081***	0.003	0.034	-0.017	0.155***	0.018	0.012
**WC**	Slope WD	-2.266	4.741***	-3.150***	4.530***	1.143***	-0.040	-1.028*	-0.102	-1.905*	-0.704	-0.426
	Interaction	7.536***	-1.010	8.477***	-7.029***	-2.833***	-0.677***	-0.932	-1.240***	0.686	-1.580*	-0.729
**Ψ**	Slope WD	-0.610***	0.402*	-0.567***	0.556***	0.217***	0.047*	-0.111	0.048	-0.394***	-0.077	-0.056
	Interaction	0.345*	-0.528**	0.152	-0.416**	-0.135***	-0.027	0.055	-0.022	0.383**	0.061	0.087

Supplemental Table S3. Top 30 wavelengths with high VIP values for each of the PLSR models. The wavelengths that correspond with wavelength regions used in the indices are bold underlined. The index most strongly correlated with gs was R775/R510 (NIR region), with Fv'/Fm' was RGRI (red region), with ΦCO2 was RVI870/610 (NIR region), with ΦPS2 was RGRI (red, green region), with A was R953/529 (NIR water absorption trough), with E was NDI1407/1862 (around 1400 and 1860 nm), with WC was WCI (NIR water absorption trough), and with Ψ was WP2 (red and red-edge region).

**Importance**	g_s	F_v'/F_m'	Φ_{CO2}	Φ_{PS2}	**A**	**E**	**WC**	**Ψ**
1	2531	2531	2531	2531	2531	2531	2531	2531
2	2525	2525	2525	2525	2525	2525	2525	2525
3	2519	2519	711	2519	2519	2519	2519	2519
4	2513	727	924	727	**943**	2513	2513	2513
5	2506	724	946	724	**924**	2506	727	727
6	727	730	1388	730	**956**	953	724	724
7	724	721	727	721	778	999	730	730
8	730	2513	730	2513	788	996	721	721
9	2500	734	**737**	734	785	**1395**	734	2506
10	721	718	1395	718	781	**1388**	2506	734
11	**734**	2506	953	2506	833	960	718	**718**
12	924	737	**740**	737	817	992	737	2500
13	943	2500	715	2500	807	989	2500	737
14	2494	715	**734**	715	775	**1401**	715	2494
15	946	2494	943	2494	852	956	2494	**715**
16	**737**	740	2519	492	**999**	924	740	740
17	718	2488	**746**	740	801	986	2488	2488
18	1395	711	1382	953	791	**1382**	743	2482
19	927	**667**	979	711	814	2500	711	743
20	940	**658**	927	2488	**989**	**1874**	2482	480
21	1388	**670**	724	495	804	983	999	486
22	2488	**654**	**743**	**505**	839	920	746	489
23	**740**	**664**	976	480	836	788	**953**	492
24	1401	**661**	**817**	1006	891	778	2476	**711**
25	920	2482	996	486	798	801	480	2476
26	930	**651**	**766**	483	849	785	**986**	**667**
27	1382	**673**	489	**676**	772	817	**996**	996
28	933	**683**	920	**670**	830	833	502	**664**
29	1013	**648**	**788**	2482	894	1013	489	483
30	**743**	**676**	2506	996	843	976	**989**	**670**
