# Supplementary material for: Proximal Hyperspectral Imaging Detects Diurnal and Drought-Induced Changes in Maize Physiology
Source: Front Plant Sci. 2021 Feb 22;12:640914. doi: 10.3389/fpls.2021.640914 (PMC7937976; doi:10.3389/fpls.2021.640914)
Supplement: Supplementary Results 1 — Maturation of maize plants affects physiology, reflectance and drought effects. [file Data_Sheet_3.PDF]

File S1: Supplemental Results

S1: Maturation of Maize Plants Affects Physiology, Reflectance and drought effects

During this study, plant leaves transitioned from a juvenile appearance (up to developmental stage V4) to mature features (from V7 onwards). Heteroblastic changes are not limited to anatomy and morphology (Kerstetter and Poethig, 1998), as water potential (ψ), transpiration rate (E) and photosynthetic rate (A) values increased (Figure 3, Supplemental Figure S2) and water content (WC) decreased in the well-watered (WW) treatment. The physiological changes coincided with a decrease in the visible (VIS) (523, 551 and 658 nm, Figure 3A) region reflectance and an increase in red-edge and near-infrared (NIR) (721 and 976 nm) reflectance. During the transition period, ψ correlated positively with red-edge and NIR reflectance (721 nm: r=0.49, 976 nm: r=0.37), while the correlation was negative with blue-green (523 nm: r=-0.49) and red (658 nm: r=-0.48) reflectance. Developmental changes in E and A were negatively related to blue-green (523 nm: r_A=-0.59, r_E=-0.50) and positively to red-edge (721 nm: r_A=0.58, r_E=0.55) and NIR (976 nm: r_A=0.69, r_E=0.74). The latter is influenced by WC, leaf thickness and anatomy (Slaton et al., 2001; Neilson et al., 2015). In addition, NIR and red-edge reflectances were also negatively correlated with WC (721 nm: r=-0.70, 976 nm: r=-0.89), which was also negatively related to shortwave infrared (SWIR) reflectance and the water absorption troughs (1694 nm: r=-0.84, 1232 nm trough: r=-0.60, 1445 nm trough: r=-0.88, 1825 nm trough: r=-0.73 and 1955 nm trough: r=-0.82). The reflectance–physiology relationships in WW plants resulted therefore from both diurnal and developmental changes.

The developmental changes of water deficit (WD) plants were less pronounced, as they reached the V6 stage two days later than the WW plants. This delay in development increased the treatment differences of Ψ, stomatal conductance (g_s), E and A, as both drought and the developmental delay caused a decrease of these traits. The additional difference caused by the developmental delay was also present in the reflectance of blue-green, red, red-edge and NIR (523, 658, 721 and 976 nm), where the drought-induced reflectance changes showed an opposite trend to the development-induced reflectance changes. One exception was green reflectance, where both drought and development caused a decrease in reflectance, resulting in reduced treatment differences at the end of the acute drought period (day 7 and 8, Supplemental Figure S3).

S2: Drought Sensitivity of New and Existing Indices

Hyperspectral indices could be grouped according to their drought sensitivity, resulting in four groups: very sensitive, sensitive, moderately sensitive and insensitive. The very sensitive group of indices showed significant drought effects from the second day of drought onwards ($P<0.05$, Supplemental Figure S5, Supplemental Figure S6) and contained those that combined red with green or red-edge reflection, such as the existing Red Green Ratio Index (RGRI), and the new Water Potential Index 1 (WPI1), Water Potential Index 2 (WPI2), Adjusted Red Green ratio Index (ARGI) and Inverse Normalized Ratio index ($IND_{715/655}$) indices. As red reflection was found to be strongly related to Ψ, effective quantum yield of photosystem 2 (Φ_{PS2}) and energy harvesting efficiency by oxidized PS2 (F_v'/F_m'), it could be used to predict these physiological traits (Figure 5). The remote sensing index RGRI showed a high prediction accuracy for all three traits, with R^2=0.78 and RMSE=0.03 for Φ_{PS2}, R^2=0.80 and RMSE=0.02 for F_v'/F_m', and R^2=0.84 and RMSE=0.12 for Ψ. However, the RGRI prediction accuracy of Ψ was outperformed by the new WPI2 index (R^2=0.92 and RMSE=0.08), which used the red-edge and a water absorption trough at 1450 nm instead of green reflectance. The prediction accuracy of the photosynthetic

efficiency traits F_v'/F_m' and Φ_{PS2} were not substantially improved by any of the new indices (Figure 5).

The sensitive group consisted of the indices Modified Chlorophyll Absorption Ratio Index (MCARI) and Carotenoid Reflectance Index 1 (CRI1), as they were able to detect treatment differences from three days after the onset of drought ($P<0.05$, Supplemental Figure S5, Supplemental Figure S6). MCARI and CRI1 were originally created to estimate chlorophyll and carotenoid content in plants, respectively, but pigment content did not differ between treatments. Instead MCARI and CRI1 were most strongly related to Ψ (Figure 5).

The ability to detect drought from the moderately sensitive group was more variable, with the first drought effects observed between the fourth and sixth day ($P<0.05$, Supplemental Figure S5, Supplemental Figure S7). These indices (Normalized Difference index 1407/1862 ($NDI_{1407/1862}$), Normalized Difference Vegetation Index (NDVI), Ratio NIR/510 ($R_{755/510}$), Ratio Vegetation Index 870/610 ($RVI_{870/610}$), Relative Water Content index (RWC), Ratio 1451/1263 ($R_{1451/1263}$), Moisture Stress Index (MSI), Water Content Index (WCI), Ratio index 953/520 ($R_{953/520}$), Ratio index 960/699 ($R_{960/699}$) and Ratio index 953/492 ($R_{953/492}$)) used NIR or SWIR reflection, which responded more slowly to drought than red reflection. Some of them were correlated with g_s, A, quantum yield based on CO2 (Φ_{CO2}), E and WC ($r_{gs\text{-}R775/510}$=0.64, $r_{A\text{-}R953/520}$=0.69, $r_{\Phi CO2\text{-}RVI870/610}$=0.7, $r_{E\text{-}NDI1407/1862}$=-0.79, $r_{WC\text{-}WCI}$=-0.87, $P<0.0001$). Simple ratios between NIR and VIS were adequate to predict Φ_{CO2}, g_s and A. Φ_{CO2} could be modeled using the existing $RVI_{870/610}$ index (prediction R^2=0.70, RMSE= 0.007) (Figure 5), which combines orange (610 nm) and NIR (870 nm) reflection. It had a treatment-independent relationship with Φ_{CO2}. The indices that performed best for A and g_s were the new $R_{953/520}$ (R^2=0.58, RMSE=1.99) and the existing $R_{775/510}$ (R^2=0.70, RMSE=0.014), respectively. These indices could detect the differences between treatments and development-induced changes in A and g_s, but were not able to predict the diurnal pattern (Supplemental Figure S2). The new normalized difference ratio index $NDI_{1407/1862}$ showed a slightly better simulation of the diurnal pattern in A but had a much lower accuracy overall (R^2=0.39, RMSE=2.553). $NDI_{1407/1862}$ was the only index that showed a relative good prediction accuracy of E (R^2=0.69, RMSE= 0.34, Figure 5, Supplemental Figure S7). It uses two SWIR wavelengths that are located in the water absorption troughs at 1450 and 1900 nm. Neither of these wavelengths were strongly correlated with E, but the ratio and normalized difference ratio showed a linear relationship with E. Most of the existing water content indices (MSI and RWC) showed a late drought effect (day 5) and were therefore present in the moderately sensitive group. None of these indices could be used to estimate the WC. RWC and $R_{1451/R1263}$ on the other hand had a strong relationship with ψ (Figure 5). A new WCI was created to predict the diurnal, developmental and drought-induced changes in WC (R^2=0.73, RMSE=0.91). It combines the NIR water absorption trough, which had a strong relationship with the WC in the WW treatment, with red and green reflectance showing WC relationships in the WD treatment. Due to the small difference in WC values between the WW and WD treatment, this index predicted significant treatment differences only from day 6 onwards (Supplemental Figure S7).

The insensitive group contained the indices Water Band Index (WBI) and Photochemical reflectance Index (PRI), which were not able to detect drought effects in this study (Supplemental Figure S5, Supplemental Figure S7). PRI was originally created to estimate a plant's photosynthetic light use efficiency by detecting changes in carotenoid content and the epoxidation state of the xanthophyll cycle pigments. No drought-induced differences in carotenoid content were found in this study, but significant changes in the light use efficiency traits F_v'/F_m' and Φ_{PS2} were oberved (Figure 5, Supplemental Figure S2). PRI showed no correlation with these latter traits. The other drought-insensitive index WBI was originally created to estimate plant water content, but showed no relationship with WC or ψ. It did show

a clear diurnal pattern with lower values in the morning compared to the afternoon. Diurnal patterns were not visible in the other WC indices (MSI and RWC), but were observed in most of the remaining indices (Supplemental Figure S5).

File S2: Supplemental Material and Methods

S1: Pigment content extraction

Anthocyanin, carotenoid and chlorophyll were extracted from two leaf discs with a diameter of 1 cm (± 50 mg). The pigment leaf samples were snap-frozen in liquid N2 and ground for extraction. Anthocyanin was extracted using the protocol of (Neff and Chory, 1998) and measured with a spectrometer at 530 nm and 657 nm. Anthocyanin content was calculated from the absorbance (abs) values using the following formula:

$$anthocyanin\ per\ mg\ fresh\ weight = \frac{(abs_{530} - abs_{657}) * 1000}{tissue\ fresh\ weight}$$

Chlorophyll and carotenoid extraction was performed using dimethyl sulphoxide (DMSO). Ground leaf samples, containing 1 ml DMSO, were heated to 65°C for 30 min in a water bath. After extraction, the samples were centrifuged and the supernatants were measured with a spectrometer at 661 nm, 643 nm, 470 nm and 534 nm. Chlorophyll and carotenoid contents (g/l) were estimated using the equations of (Sims and Gamon, 2002):

$$anthocyanin = 0.082 * abs_{534} - 0.00687 * abs_{643} - 0.002423 * abs_{661}$$
$$chlorophyll\ (chl\beta) = 0.02255 * abs_{643} - 0.00439 * abs_{534} - 0.004488 * abs_{661}$$
$$chlorophyll\ (chl\alpha) = 0.01261 * abs_{661} - 0.001023 * abs_{534} - 0.0022 * abs_{643}$$
$$carotenoids = \frac{[abs_{470} - 17.1 * (chl\alpha + chl\beta) - 9.479 * anthocyanin]}{119.26}$$

The chlorophyll and carotenoid concentrations were converted to mg per g fresh weight.

S2: Statistics

To determine drought effects on the whole plant and light class population averages for each time point (first experiment: 8 time points over 8 days, second experiment: 18 time points over 4 days) a mixed model analysis was performed on the longitudinal reflectance data with the MIXED procedure of the SAS software (Version 9.4 of the SAS System for windows, SAS Institute Inc., Cary, NC, USA) for each of the 11 wavelengths. The dependent variable of this model was reflectance and the fixed effects were treatment, time and their interaction term. Time was treated as a factor in these models and corresponded with day for the first experiment, while it was a concatenation of day and time-of-day for the second experiment.

$$reflectance = treatment + time + interaction \qquad \text{model 1}$$

A Toeplitz covariance structure was used to model the correlations between reflectance measurements of the same plant. From this model, the least-squares means estimates for the interaction term were saved. Subsequently, a mixed model was fitted to the gas exchange measurements that were done at the same time points. The fixed effects part of this model was the same as for the reflectance data and the same covariance structure was used. To find associations between the non-destructive physiological traits and reflectance for the whole experiment, a mixed model was used that related the physiological trait with reflectance of the 11 wavelengths, hereby taking treatment and the interaction between treatment and reflectance into account (model 2 and model 3). Input data for reflectance and physiological trait were the previously estimated population averages.

$$physiological\ trait = treatment + reflectance + interaction \qquad \text{model 2}$$

When the interaction term was not significant at the 5 % significance level, an additive model was fitted:

$$physiological\ trait = treatment + reflectance \qquad \text{model 3}$$

Drought treatment effects on the destructively measured physiological traits (Ψ, WC, pigment content) were analyzed in a similar way as the non-destructive gas exchange measurements. As no repeated measures were available for these traits, linear models were fitted with the lm function ('stats' R package). The dependent variable of these models was the trait of interest and the independent variables were treatment, the concatenated time variable and their interaction. The least-square means estimates ('lsmeans version 2.27-62' R package) of the interaction term were calculated and the treatment differences were evaluated by calculating contrasts for each time point. The p-values were corrected for multiple testing with the Sidak correction. The associations between the destructive measurements and reflectance for the whole experiment were determined with a linear model that accounted for the difference in heterogeneity between WW and WD plants by estimating different variances for the treatments ('nlme' package). Two types of models were developed: the first model related the physiological trait with reflectance (model 2), while the second model also took treatment and the interaction between treatment and reflectance into account (model 3).
The exploratory experiment (EXP) showed variation that corresponded with the time of imaging. To test this time effect and how it differed between wavelengths, wavelength absorption trough depths and treatments, a linear model was created for the sixth day after the drought initiation. This model had reflectance as dependent variable, the continuous variable time, a combined treatment/wavelength (or treatment/wavelength trough) variable and their interaction as independent variables. The model was created with the 'nlme' R package and incorporated heterogeneous variances for the treatment/wavelength variable (Pinheiro et al., 2017).

$$reflectance\ day\ 6 = time + treatment/wavelength + interaction \ \text{model 4}$$

The slopes of this model and contrasts of treatments for each wavelength, wavelengths and wavelength troughs for each treatment were calculated using the 'lsmeans' R package (Lenth, 2016). The resulting p-values were corrected using the conservative sequential goodness-of-fit multiple testing method ('SGoF' function of the 'sgof' R package,(Castro-Conde and de Uña-Álvarez, 2016). To incorporate the time-of-day effect into model 1 of EXP, the treatment differences for the intermediate light class were reanalyzed by incorporating a time-grouping factor. Plants were grouped in time intervals of 1 hour and treatment differences were tested within each hour for each day (nWW: 8 ± 4 ,nWD: 8 ± 3) with a factorial ANOVA using the 'lsmeans' package. A two-stage multiple testing correction as described by Van den Berge et al. (2017) was applied using False Discovery Rate and Sidak correction with the 'lsmeans', 'MHTdiscrete' and 'stats' package (Van den Berge et al., 2017). The aforementioned factorial ANOVA and two-stage p-value correction were also used to determine the significant treatment differences for the indices. To relate diurnal changes in physiology to reflectance, a correlation analysis was performed with day or a day-pot number variable as random factor ('rmcorr' and 'stats' R package)

References

Castro-Conde, I., and De Uña-Álvarez, J. (2016). sgof: multiple hypothesis testing. R package version 2.3 (https://CRAN.R-project.org/package=sgof).

Kerstetter, R.A., and Poethig, R.S. (1998). The specification of leaf identity during shoot development. Annual Review of Cell and Developmental Biology 14, 373-398.

Lenth, R. (2016). Least-squares means: the R package lsmeans. Journal of Statistical Software 69, 1–33.

Neff, M.M., and Chory, J. (1998). Genetic interactions between phytochrome A, phytochrome B, and cryptochrome 1 during Arabidopsis development. Plant Physiology 118, 27-36.

Pinheiro, J., Bates, D., Debroy, S., Sarkar, D., and R Core Team (2017). nlme: linear and nonlinear mixed effects models. R package version 3.1-131 (https://CRAN.R-project.org/package=nlme).

Sims, D.A., and Gamon, J.A. (2002). Relationships between leaf pigment content and spectral reflectance across a wide range of species, leaf structures and developmental stages. Remote Sensing of Environment 81, 337-354.

Van Den Berge, K., Soneson, C., Robinson, M.D., and Clement, L. (2017). stageR: a general stage-wise method for controlling the gene-level false discovery rate in differential expression and differential transcript usage. Genome Biology 18, 151.
